# Genomes of the rice pest brown planthopper and its endosymbionts reveal complex complementary contributions for host adaptation

**DOI:** 10.1186/s13059-014-0521-0

**Published:** 2014-12-03

**Authors:** Jian Xue, Xin Zhou, Chuan-Xi Zhang, Li-Li Yu, Hai-Wei Fan, Zhuo Wang, Hai-Jun Xu, Yu Xi, Zeng-Rong Zhu, Wen-Wu Zhou, Peng-Lu Pan, Bao-Ling Li, John K Colbourne, Hiroaki Noda, Yoshitaka Suetsugu, Tetsuya Kobayashi, Yuan Zheng, Shanlin Liu, Rui Zhang, Yang Liu, Ya-Dan Luo, Dong-Ming Fang, Yan Chen, Dong-Liang Zhan, Xiao-Dan Lv, Yue Cai, Zhao-Bao Wang, Hai-Jian Huang, Ruo-Lin Cheng, Xue-Chao Zhang, Yi-Han Lou, Bing Yu, Ji-Chong Zhuo, Yu-Xuan Ye, Wen-Qing Zhang, Zhi-Cheng Shen, Huan-Ming Yang, Jian Wang, Jun Wang, Yan-Yuan Bao, Jia-An Cheng

**Affiliations:** State Key Laboratory of Rice Biology/Institute of Insect Science, Zhejiang University, Hangzhou, 310058 China; BGI-Shenzhen, Shenzhen, 518083, Guangdong China; BGI-Tech, BGI-Shenzhen, Shenzhen, 518083, Guangdong China; School of Biosciences, The University of Birmingham, Birmingham, B15 2TT United Kingdom; National Institute of Agrobiological Sciences, Tsukuba, 305-8634 Japan; State Key Laboratory for Biocontrol/Institute of Entomology, Sun Yat Sen University, Guangzhou, 510275 China; Department of Biology, University of Copenhagen, Ole Maaløes Vej 5, 2200 Copenhagen, Denmark; Princess Al Jawhara Center of Excellence in the Research of Hereditary Disorders, King Abdulaziz University, Jeddah, 21589 Saudi Arabia; China National GeneBank, BGI-Shenzhen, Guangdong, 518083 China; James D. Watson Institute of Genome Science, Hangzhou, China; Macau University of Science and Technology, Avenida Wai long, Taipa, Macau, 999078 China; Department of Medicine, University of Hong Kong, Pokfulam, Hong Kong

## Abstract

**Background:**

The brown planthopper, *Nilaparvata lugens*, the most destructive pest of rice, is a typical monophagous herbivore that feeds exclusively on rice sap, which migrates over long distances. Outbreaks of it have re-occurred approximately every three years in Asia. It has also been used as a model system for ecological studies and for developing effective pest management. To better understand how a monophagous sap-sucking arthropod herbivore has adapted to its exclusive host selection and to provide insights to improve pest control, we analyzed the genomes of the brown planthopper and its two endosymbionts.

**Results:**

We describe the 1.14 gigabase planthopper draft genome and the genomes of two microbial endosymbionts that permit the planthopper to forage exclusively on rice fields. Only 40.8% of the 27,571 identified *Nilaparvata* protein coding genes have detectable shared homology with the proteomes of the other 14 arthropods included in this study, reflecting large-scale gene losses including in evolutionarily conserved gene families and biochemical pathways. These unique genomic features are functionally associated with the animal’s exclusive plant host selection. Genes missing from the insect in conserved biochemical pathways that are essential for its survival on the nutritionally imbalanced sap diet are present in the genomes of its microbial endosymbionts, which have evolved to complement the mutualistic nutritional needs of the host.

**Conclusions:**

Our study reveals a series of complex adaptations of the brown planthopper involving a variety of biological processes, that result in its highly destructive impact on the exclusive host rice. All these findings highlight potential directions for effective pest control of the planthopper.

**Electronic supplementary material:**

The online version of this article (doi:10.1186/s13059-014-0521-0) contains supplementary material, which is available to authorized users.

## Background

**Figure 1:**
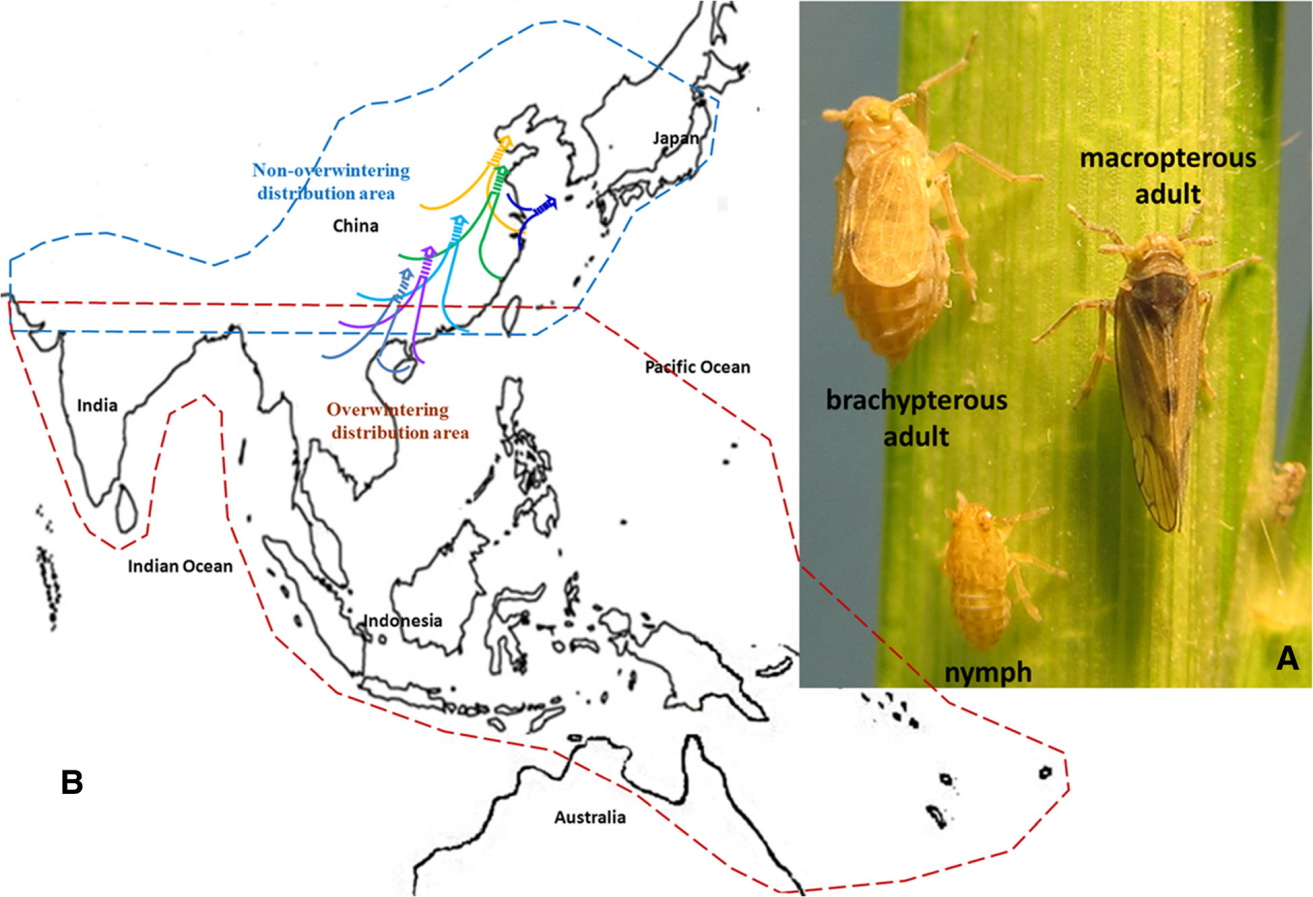
**The brown planthopper. (A)** Short-winged (brachypterous) and long-winged (macropterous) female adults and nymph. **(B)** Schematic diagram of BPH distribution worldwide and possible northward migratory routes (arrows) in East China.

The brown planthopper (BPH), *Nilaparvata lugens* (Stål) (Hemiptera: Delphacidae) (Figure 1A), has become the most destructive pest for rice (*Oryza sativa*) - the major food source for half of the world’s population - since Asian farmers adopted green revolution technologies in the 1960s, that is, agricultural practices using genetically improved cultivars, synthetic fertilizers and pesticides [1]. BPH is equipped with special biological features that enable frequent outbreaks of it in condensed rice paddy fields, which have been used continuously for monoculture across large areas of Asia, under heavy use of nitrogen fertilizer and insecticides. Features contributing to the success of the insect include its mystical capacity to live on a sole host plant and to overcome host plant resistance, association with multiple endosymbionts, high fecundity, and long distance migration (Figure 1A,B). Although various new rice varieties with high resistance to BPH and new insecticides, as well as integrated pest management (IPM) programs, have been developed and implemented, Asian countries have continually experienced serious outbreaks of BPH in the new century. Approximately 10 to 20 million hectares of rice fields were destroyed by BPH through direct sucking and transmittal of ragged stunt virus and grassy stunt virus in 2005 [2,3].

As a monophagous insect, BPH feeds only on the phloem sap of the rice plant and can also quickly overcome resistance genes in its rice host through the development of new virulence [4]. BPHs occur as a complex of populations that exhibit varying abilities to survive on and infest host varieties possessing different resistance genes [5,6]. Furthermore, recent studies have revealed that the yeast-like endosymbionts (YLS) and bacterial symbionts of this insect might also play important roles in insect-plant competition [7,8], thereby forming an integrative part of BPH’s adaptation to its rice host.

Among all the features of BPH, perhaps the best known are its wing dimorphism (macropterous and brachypterous adults) and capability for long-distance migration, which enable it to exploit its exclusive rice host in temperate regions and results in maximized damage to rice production across wide geographic areas (Figure 1B). The northern geographic limit of winter breeding for BPH is approximately 23 to 25°N, and it does not survive winter in temperate regions [9]. Migratory macropterous adults remain in reproductive diapause after emergence, but their capacity for long-distance flight enables them to migrate northward when rice becomes available in temperate areas of China, northern India, Japan, and Korea [10]. During autumn, returning migrations (north-to-south) of BPH populations have been observed across China and India [11]. However, most adults of subsequent generations post-migration are brachypterous, exhibiting increased fecundity due to shortened pre-oviposition and extended oviposition periods [12]. When the local habitat deteriorates critically, such as when host rice matures, the fitness of the brachypterous morph plummets because of its inability to escape and colonize more favorable habitats elsewhere. Consequently, the macropterous morph becomes dominant [12].

In this study, we obtained a 1.14 Gbp draft BPH genome and identified 27,571 protein-coding genes. The BPH genome is the first characterized genome of a monophagous sap-sucking arthropod herbivore. In addition, we sequenced the genomes of both the YLS and bacterial endosymbionts of BPH. By targeted investigations of these three genomes with reference to the increasing diversity of arthropod genomes, we discovered that components in the chemoreception, detoxification, and digestive enzyme gene families in the BPH genome have contracted or been lost. However, genes essential for BPH’s survival on rice sap, a nutritionally imbalanced food source, were discovered within the genomes of its microbial endosymbionts, which have evolved to complement the needs of their host.

## Results and discussion

### Assembly of a large and complex genome using combined whole-genome shotgun and pooled fosmid sequencing

The BPH genome size is estimated to be approximately 1.2 Gbp using a k-mer approach, which is consistent with our previous estimates using flow cytometry. However, analysis of 17-mers suggest higher than expected heterozygosity of the BPH genome despite 13 generations of inbreeding, which presents a challenge for *de novo* genome assembly when using only whole-genome shotgun (WGS) sequence data. To overcome the challenge caused by high heterozygosity and repeat sequence content, we employed a hybrid method that integrates WGS sequences with pooled fosmid sequencing. The TrimDup module in Rabbit was used to remove redundant and heterozygous sequences [13]. Finally we assembled a draft BPH genome of 1.14 Gbp, with a scaffold N50 of 356.6 kbp and a contig N50 of 24.2 kbp (Table 1). We evaluated the completeness of the draft genome assembly by mapping expressed sequence tags (ESTs) to the genome and by calculating coverage for a set of 248 core eukaryotic genes using CEGMA [14], which show genome coverage rates of 97.1% and >96%, respectively (Tables S1 to S8 and Figures S1 to S5 in Additional file 1).

**Table 1:** **Features of the assembled genomes and gene sets of** ***Nilaparvata lugens*** **and another hemipteran insect,** ***Acyrthosiphon pisum***

	***N. lugens***	***A. pisum***
Assembled genome size (Mb)	1,141	464
Estimated size (based on k-mer analysis, Mb)	1,220	
Number of chromosomes	30	4
Contig N50/scaffold N50 (kbp)	24.2/356.6	11.8/86.9
**Assembly evaluation (covered by assembly**)		
ESTs (%)	95.6	99
CEGMA genes (%)	96.8	100
**Genomic features**		
Repeat (%)	48.6	33.3
G + C (%)	34.6	29.6
Coding (%)	2.74	6.45
Intron (%)	25.3	24.6
Number of tRNAs	2,630	-
**Gene repertoire**		
Number of protein-coding genes	27,571	33,267
with InterPro domains	12,734	13,878
with Gene Ontology terms	10,245	10,980
Species-specific genes	16,330	19,586

### BPH has a large genome with highly repetitive content and a large number of species-specific genes

Given the large and complex nature of the BPH genome, we analyzed the genome structure and carried out detailed annotation along with comparison with other insect species.

#### G + C content

The BPH genome is A + T rich, exhibiting only 34.6% G + C, which is higher than that of the only published hemipteran species, the pea aphid (*Acyrthosiphon pisum*; 29.6%) [15], and lower than that of *Drosophila melanogaster* (42%) [16]. We compared the G + C content distribution and sequencing depth of BPH and four other insect species, and found that BPH showed a similar distribution pattern to that of the pea aphid (Figures S6 and S7 in Additional file 1).

#### Repetitive sequences

A significant proportion of the BPH genome contains a high level of repetitive sequences (48.6%, including tandem repeats and transposable elements), which is a larger fraction than that measured in the pea aphid (33.3%) [15]; tandem repeats account for 6.4% of the whole genome. Transposable elements (TEs) were identified at both the DNA and inferred protein level. The TEs account for approximately 38.90% of the BPH genome, including DNA repeats (14.2%), long interspersed nuclear elements (LINEs; 16.0%), long terminal repeats (LTRs; 14.8%), short interspersed nuclear elements (SINEs; 0.7%), and unknown repeat types (1.9%). Comparison of TEs identified through homology-based and *de novo* prediction approaches against those from Repbase revealed a shift of the peak sequence divergence ratio. This finding suggests that the BPH-specific TEs, especially DNA transposons, have evolved relatively recently, and likely contribute to the large genome size of BPH (Tables S9 and S10 and Figure S8 in Additional file 1).

#### Gene annotation

**Figure 2:**
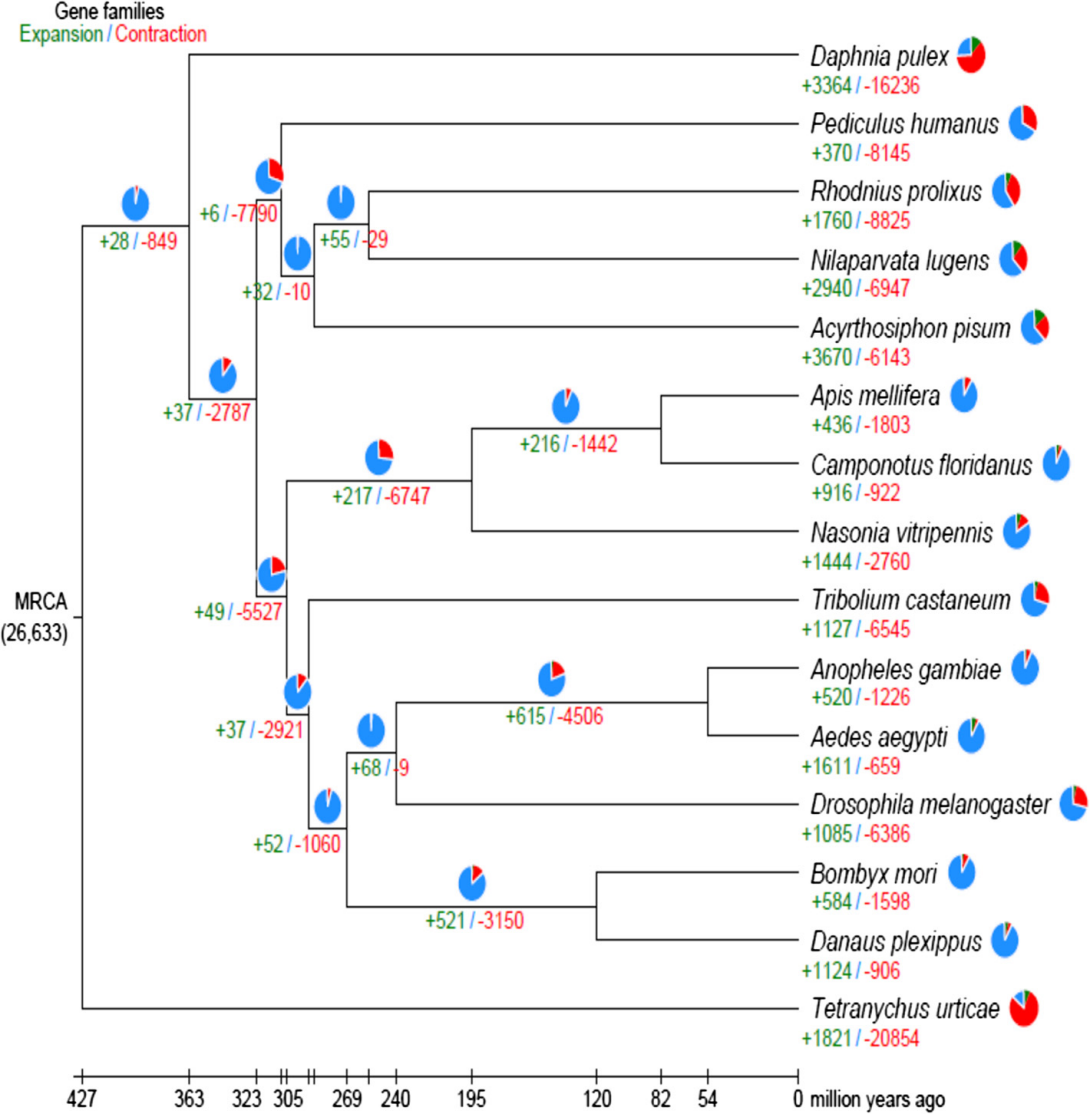
**Gene family expansions and contractions in the brown planthopper compared with other arthropod genomes.** Numbers for expanded (green) and contracted (red) gene families are shown below branches or taxon names with percentages indicated by pie charts.

We predicted protein-coding genes using GENEWISE [17], an homology-based method referring to protein sequences from four representative insects and from human. We also used the programs GENSCAN [18] and AUGUSTUS [19] for additional *ab initio* gene predictions. These results were then combined using GLEAN to generate a consensus gene set [20]. A 2.47 Gbp RNA-seq data set was additionally used to complement the combined gene set. Finally, we created a reference gene set containing 27,571 protein-coding genes for BPH. Among the 15 arthropod genomes compared in this study, the numbers of predicted genes and species-specific genes in BPH were lower than in the pea aphid (Table 1), but higher than those of most other insects. The lack of accumulated knowledge on arthropod genomes in general might have attributed to the elevated species-specific gene components in BPH because sequenced arthropod genomes are limited and highly biased in phylogenetic coverage. For instance, the first sequenced crustacean, the waterflea (*Daphnia pulex*), has an unexpected proportion of unknown genes (36% of over 30,000 genes) when compared with other genomes [21]. Similarly, as most published insect genomes are from holometabolans, 37% of the predicted genes in the pea aphid (the first basal hemimetabolous species with sequenced genome) could not be matched to any known species [15]. Although the likelihood of overestimation during gene predictions cannot be completely ruled out, the pattern of elevated proportions of species-specific genes is shared among all hemipteran genomes (*A. pisum*, *Rhodnius prolixus*, and *N. lugens* (Figure 2). We expect that a higher level of homology can be discovered when additional genomes are sequenced for more hemipteran insects.

Although the functions of 40.5% of the BPH genes remain unidentified when compared with proteins in existing databases (‘unannotated genes’; Tables S11 to S13 in Additional file 1), most of them are expected to be correctly assembled with support from expressed RNA data and RT-PCR results. For example, 30.41% of unannotated genes were indeed expressed (at 98% identity threshold; Table S14 in Additional file 1). Furthermore, we randomly chose 30 unannotated genes among those with RNA sequence support (Table S15 in Additional file 1) for RT-PCR and sequencing analysis. Twenty-four predicted full coding sequences (CDSs) were successfully amplified, while six CDSs failed to be amplified (Figure S9 in Additional file 1). Additionally, 20 PCR products were cloned and sequenced. The sequencing results confirmed the accuracy of the sequence assembly, although some genes might contain varying numbers of exons or differ in length. However, a large proportion (69.59%; Table S14 in Additional file 1) of unannotated genes have no transcript evidence at present and the likelihood of over-annotation remained after the implementation of two hidden Markov model-based *de novo* prediction programs, *Angustus* and *Genescan*. The gene length distribution of the entire BPH gene set, DNA CDSs, and exons are generally similar to those observed in *Tribolium castaneum* [22], *Apis mellifera* [23], and *A. pisum* [15], but BPH has a larger average intron length (281.41 Mbp) compared with that in *A. pisum* (114.06 Mbp), which likely also contributs to the large BPH genome size (Table 1; Figure S10 in Additional file 1).

We annotated four categories of non-coding RNA (ncRNA) in the BPH genome: microRNA (miRNA), transfer RNA (tRNA), ribosomal RNA (rRNA), and small nuclear RNA (snRNA) (Table S16 in Additional file 1).

#### Gene orthology and phylogenomics

**Figure 3:**
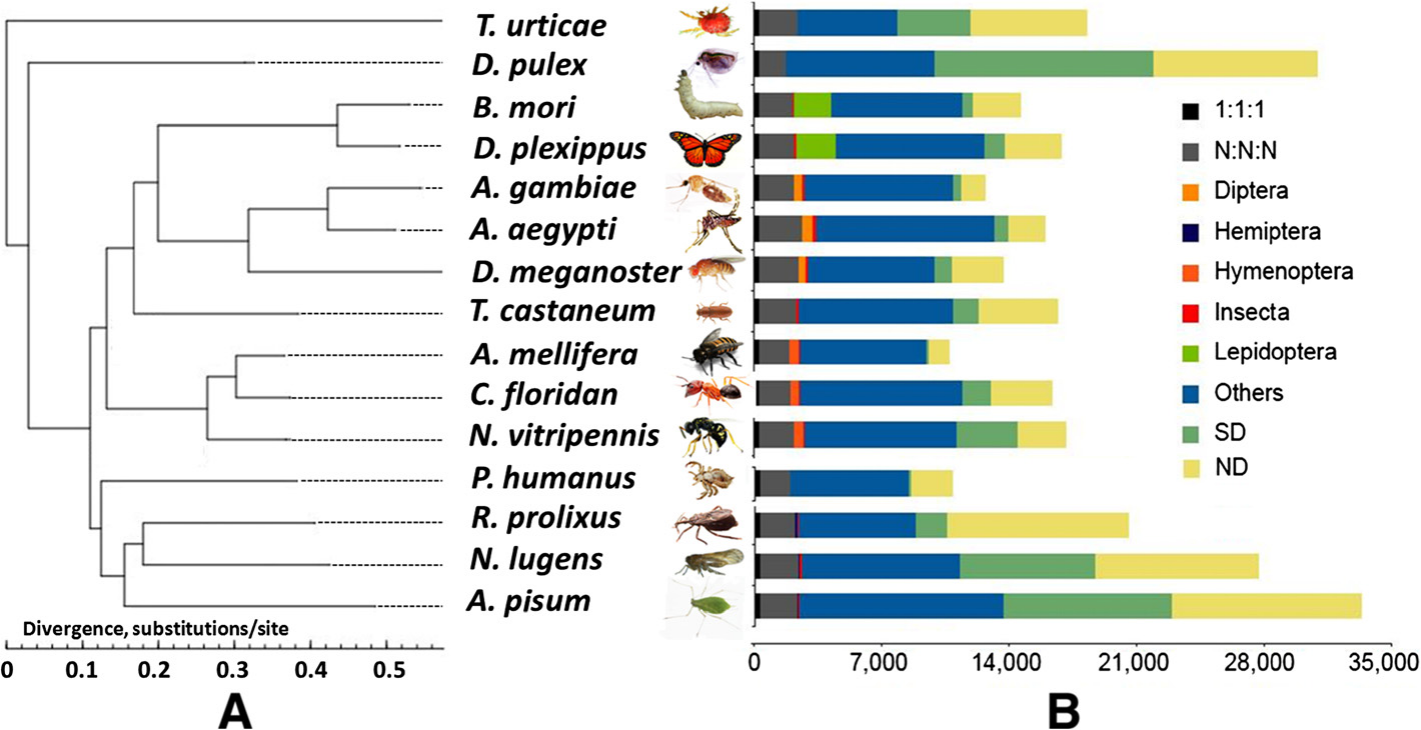
**Phylogenetic relationships and gene orthology based on the genomes of 15 arthropod species. (A)** phylogenetic relations of BPH to insects and other arthropods based on single-copy orthologous genes obtained from full genomes. Thirteen insect species were used for the analysis, including *Bombyx mori*, *Danaus plexippus*, *Anopheles gambiae*, *Aedes aegypti*, *Drosophila melanogaster*, *Tribolium castaneum*, *Apis mellifera*, *Camponotus floridanus*, *Nasonia vitripennis*, *Pediculus humanus*, *Rhodnius prolixus*, *Nilaparvata lugens*, and *Acyrthosiphon pisum*. Two Arthropoda animals (*Daphnia pulex*, *Tetranychus urticae*) were used as outgroup taxa. Branch lengths represents divergence times estimated by second codon positions of 318 single-copy genes (Table S17 in Additional file 1) using *PhyML* [80,81], with a gamma distribution across sites and an HKY85 substitution model. The branch supports were inferred based on approximate likelihood ratio test (aLRT) **(B)** Gene orthology comparison among the genomes of 15 arthropod species. Note: the order of the 15 species follows that in Figure 3A. 1:1:1 refers to single-copy gene orthologs found across all 15 lineages. N:N:N refers to multi-copy gene paralogs found across all 15 lineages. Diptera, Lepidoptera, Hymenoptera, Hemiptera, and Insect refer to taxon-specific genes that are present only in the relevant lineage. SD indicates species-specific genes in multiple copies. ND indicates species-specific genes in single copies*.*

We compared the annotated BPH genes with those of 12 insect and 2 other arthropod species with annotated genome sequences publically available, and identified 16,330 Treefam-method-defined BPH species-specific genes (59% of the whole gene set; Table 1, Figure 3B; Table S17 and Figure S11 in Additional file 1), indicating that only 40.8% of the 27,571 identified *Nilaparvata* protein-coding genes share detectable homology with other available Arthropoda proteomes. We also detected 2,421 conservative genes among all of the 15 genomes examined, including 318 strict single-copy orthologous genes (Table S17 in Additional file 1). A phylogenetic reconstruction using all 318 orthologs (of which the second codon positions comprise 219,780 sites) across the 15 arthropod taxa (Figure 3A; Figure S12 in Additional file 1) reveals that BPH is a sister taxon to the true bug *R. prolixus*, and together form a sister lineage to the pea aphid. The evidence for this relationship supports a monophyletic Hemiptera while rejecting ‘Homoptera’ [24].

Both the percentage of species-specific genes and the total gene number of all three hemipteran genomes are higher than for other insect genomes, indicating an overall expansion of the gene repertoire in species of the hemipteran lineage (Figure 2; Tables S18 and S19 in Additional file 1). The expanded genes are over-represented in Gene Ontology terms including sodium channel activity, zinc ion binding, helicase activity, and so on, whereas gene losses are most pronounced in detoxification and digestion genes, including P450, GST, and chymotrypsin gene families (Tables S20 and S21 in Additional file 1). These lineage-specific gene expansions and contractions (or losses) likely reflect the evolutionary adaptation of hemipteran insects to plant phloem sap as their food sources and their symbiotic dependencies.

### Chemoreception systems specialized for rice specificity

Chemoreception is essential for herbivorous insects in host plant selection [25]. Four multi-gene families are involved in the chemoreceptor system: odorant binding proteins (OBPs), chemosensory proteins (CSPs), odorant receptors (ORs), and gustatory receptors (GRs). In the BPH genome, we identified 11 genes coding for OBPs, 17 for CSPs, 50 for ORs, and 10 for GRs.

We compared the chemoreception gene family numbers in BPH with those of nine other insects (Table 2). Among the chemoreceptors, OBPs and CSPs are signal binding proteins that perceive chemical cues from ambient environments. Both BPH and the pea aphid have a lower number of OBPs compared with other insects.

**Table 2:** **Numbers of validated peripheral chemoreception genes in insect genomes**

		**Insect order**									
		**Diptera**				**Hymenoptera**		**Coleoptera**	**Lepidoptera**	**Hemiptera**	
**Function**	**Gene family** ^**a**^	***D.m***	***A.g***	***A.a***	***C.p***	***A.m***	***N.v***	***T.c***	***B.m***	***A.p***	***N.l***
Chemosensory	OBP	52	81	66	53	21	90	49 (1)	43 (1)	14 (1)	11
	CSP	4	8	-	-	6	-	19 (1)	19 (2)	10 (1)	17
Olfactory	OR	62	79	131	180	170	301	341	48	69 (10)	50
Gustatory	GR	68	76	91 (23)	123	10	58	63	65	75 (2)	10
Food range		P	P	P	P	P	P	P	O	O	M
Food and host		Fruits, plant wrack	Nymph: bacteria, algae; male: plant juice; female: animal blood			Honey, pollen	Fly species	Grain, oilseed	Mulberry, *Morus rubra*, *M. nigra*, Osage Orange	Legumes, Fabaceae	Rice
Mouthpart		Licking	Chewing Sucking			Chewing-sucking		Chewing	Chewing	Sucking	

^a^OBP, odorant binding protein; CSP, chemosensory protein; OR, odorant receptor; GR, gustatory receptor. Numbers represent putative functional genes and pseudogenes (in parentheses when available). Abbreviations for food ranges: P, polyphagous insect; O, oligophagous insect; M, monophagous insect. Abbreviations of insect species: *D.m*, *Drosophila melanogaster*; *A.g*, *Anopheles gambiae*; *A.a*, *Aedes aegypti*; *C.p*, *Culex quinquefasciatus*; *A.m*, *Apis mellifera*; *N.v*, *Nasonia vitripennis*; *T.c*, *Tribolium castaneum*; *B.m*, *Bombyx mori*; *A.p*, *Acyrthosiphon pisum*; *N.l*, *Nilaparvata lugens*.

OBP and CSP genes in BPH are represented in all major clades of phylogenetic trees constructed for these multi-gene families across varied insects, and many of the genes within these two families are most closely related to those of other hemipteran species, indicating conservation among all insects (Figure S13 and S14 in Additional file 1). Although the specific functions of many OBP and CSP genes in BPH are unknown, earlier studies suggested their tight associations with plant volatile reception in other systems [26-29]. Previous studies on gene function, coupled with our observation of the conserved evolution of OBPs and CSPs in hemipterans, indicate there is a fundamental involvement of chemo-signal binding genes in the recognition of plant volatiles, which is crucial for host detection. Most tested OBP and CSP genes in this study are highly expressed in antennae and/or head of BPH (Figures S15 and S16 in Additional file 1), while many CSPs were also highly or even preferably expressed in legs (for example, *NICSP16*), implying an involvement of legs in olfactory detection, or an alternative function for these tissue-specific CSPs.

Chemoreceptor genes, including ORs and GRs, convert chemical signals into neuronal activity and thus play key roles in local adaptation and reproductive isolation in insects. Their diversity in insects allows these receptors to bind a variety of ligands. Our comparison of these chemoreceptor genes from different insects (Table 2) shows that polyphagous insects have a richer diversity of chemoreceptor genes, especially those that code for olfactory receptors. Polyphagous insects (with the exception of *D. melanogaster* and *A. gambiae*) have more than twice the number of OR genes as oligophagous and monophagous insects. We also noted that the ORs show significant species-specific expansion in many of the insect species examined (Figure S17 in Additional file 1), which could be due to evolutionary adaptation of taxonomic lineages to ecological variations.

BPH has a substantially lower number of GR genes (10 versus 77 in *A. pisum*, another hemipteran species), reflecting its strict monophagous diet of rice phloem sap. This finding is consistent with results from previous analyses of chemoreception genes in 12 *Drosophila* species, where specialist species show an accelerated rate of GR gene loss compared with generalist species [30]. The BPH GR genes are represented in all major clades of phylogenetic trees constructed for this gene family across varied insects (Figure S18 in Additional file 1) and may be involved in regulation of a wide range of processes relevant to feeding behaviors.

### Specialized detoxification and digestion genes for a rice sap food source

The genes encoding cytochrome P450 monooxygenases (P450s) and glutathione S-transferases (GSTs) are members of the major multigene enzyme families that are primarily responsible for detoxification of xenobiotic compounds, for example, insecticides and plant secondary metabolites [31,32]. The expansion and contraction of these gene families in insects seem to be correlated with the levels of exposure to external stressors, for example, host defense systems and environmental toxins. A comparison of insect species with available genomes (Table 3) reveals that *N. lugens* has much fewer P450 genes (67) compared with other insects; it is ranked second to *A. mellifera* (which has 46) in having the fewest genes. The genes encoding cytochrome P450 monooxygenases, including *CYP6a2*, *CYP6a13*, *CYP6a14*, *CYP6k1* and *CYP3A18*, which belong to the CYP4 subfamily, have all been lost by *N. lugens*. It has been suggested that the relatively low number of P450 genes in honeybee may be a consequence of the social organization of the beehive, which probably shields the queen and larvae from environmental exposure to toxins, permitting a subsequent loss of P450 genes in this species [33]. The comparison of the genome data also shows that *N. lugens* and *A. mellifera* have the least number of GSTs among all sequenced insects, only half that in *A. pisum* (Table 3). Interestingly, the plant sap-sucking but polyphagous *A. pisum* possesses more P450 and GST genes, which is congruent with its need to detoxify compounds from hundreds of host species.

**Table 3:** **Comparison of detoxification and digestion genes in insect genomes**

	**Diptera**				**Hymenoptera**		**Coleoptera**	**Lepidoptera**	**Hemiptera**	
	***D.m***	***A.g***	***A.a***	***C.q***	***A.m***	***N.v***	***T.c***	***B.m***	***A.p***	***N.l***
**Detoxification P450**										
CYP2	6	10	9	15	8	6	8	8	10	10
CYP3	36	42	92	92	28	47	70	31	33	18
CYP4	32	45	68	83	4	29	44	29	32	27
Mito	11	9	10	12	6	7	9	13	8	12
**Total**	**85**	**106**	**179**	**202**	**46**	**89**	**131**	**81**	**83**	**67**
**Detoxification GST**										
Delta	11	14	10	28	2	5	3	4	10	2
Epsilon	14	8	8	1	0	0	19	4	0	1
Omega	5	1	1	1	2	2	4	4	2	1
Sigma	1	1	1	2	4	8	7	2	6	3
Theta	4	2	4	4	1	3	1	1	2	1
Zeta	2	1	1	0	1	1	1	2	0	1
Micrsomal	1	3	1	5	1	0	1	1	2	2
Unknown	0	3	3	0	0	0	0	2	0	0
**Total**	**38**	**33**	**29**	**41**	**11**	**19**	**36**	**20**	**22**	**11**
**Digestion**										
Trypsin	96	103	120	122	33	84	44	56	28	34
Chymotrypsin	56	78	64	74	7	53	60	28	6	10
Carboxypeptidase	49	60	64	64	26	38	60	58	39	42
Lipase	61	41	91	86	22	55	53	72	41	39
Alpha-amylase	3	4	9	13	1	3	12	3	0	0
Starch phosphorylase	2	2	1	1	1	1	2	1	3	2
**Total**	**267**	**288**	**349**	**360**	**90**	**234**	**231**	**218**	**115**	**127**

Detoxification- and digestion-related genes in several insect species with available genomes were investigated for *Acyrthosiphon pisum* [34], *Drosophila melanogaster* [35], *Apis mellifera* [36], *Anopheles gambiae* [37], *Tribolium castaneum* [38], *Bombyx mori* [39], *Culex quinquefasciatus* [40], *Aedes aegypti* [40], and *Nasonia vitripennis* [41]. *D.m*, *Drosophila melanogaster*; *A.g*, *Anopheles gambiae*; *A.a*, *Aedes aegypti*; *C.q*, *Culex quinquefasciatus*; *A.m*, *Apis mellifera*; *N.v*, *Nasonia vitripennis*; *T.c*, *Tribolium castaneum*; *B.m*, *Bombyx mori*; *A. p*, *Acyrthosiphon pisum.*

The genome-wide comparison also shows that many digestion-related protease and lipase genes may have undergone a major expansion in Diptera, Lepidoptera, and Coleoptera but not in Hymenoptera or Hemiptera (Table 3). *A. mellifera* has the fewest protease and lipase genes, which may reflect its colonial feeding strategy that alleviates pressure on the digestion system for individual insects. When compared with Diptera, Lepidoptera and Coleoptera, both hemipteran species have lost a significant number of genes related to digestion, including 65 trypsin, 41 chymotrypsin, 23 lipase and 12 carboxypeptidase genes (Table 3; Table S21 in Additional file 1), which can be attributed to the much reduced requirements for digesting a simple diet such as phloem sap. In addition, the *N. lugens* and *A. pisum* genomes encode sucrase and maltase genes but have lost the genes encoding the typical alpha-amylase, an enzyme that is indispensable in the digestion of starch molecules. This result is consistent with the use of plant phloem sap as their food source, which lacks starch but is rich in sugar. In contrast, the leaf-feeding silkworm and polyphagous dipteran insects have three to four alpha-amylases, while *T. castaneum*, a grain-feeding insect, has the most abundant alpha-amylases among these insect species.

Genes encoding peritrophins, the main structural proteins forming the peritrophic matrix (PM), and the chitin synthase CHS2, which is responsible for PM chitin synthesis, are lost in the BPH genome (Table S22 in Additional file 1). The PM is a chitin and glycoprotein layer that lines the midgut of most insect species, protecting the midgut epithelium from damage caused by abrasive food particles, digestive enzymes, and pathogens infectious via ingestion. This characteristic loss of PM-forming genes in BPH and *A. pisum* is also a genomic feature consistent with their plant sap diet.

### Mutualistic genomes adapted to rice host specialization

Many hemipteran insects are known to host obligate or facultative microbial endosymbionts, most of which are bacteria [42,43]. BPH harbors in its fat body cells a YLS, a filamentous ascomycete fungus belonging to the family Clavicipitaceae [44] (Figure S19 in Additional file 1). This YLS is maternally transmitted via eggs through transovarial infection and are found in every developmental stage of BPH [45]. Artificial diet-based studies indicate critical involvements of the YLS in amino acid and sterol biosynthesis, and in the recycling of nitrogen products of the BPH host [46,47], and partial amino acid biosynthesis pathways have been constructed based on transcriptome data [48]. However, the genomic mechanisms of these mutualistic interactions have not been identified. Furthermore, BPH also often hosts a facultative bacterium that is phylogenetically close to the genus *Arsenophonus*, the male-killing bacterium described in *Nasonia* [49]. The interactions of these bacteria with the host, however, are not known.

To investigate these relationships, we isolated the YLS from BPH tissues and conducted separate WGS sequencing for this fungus. The YLS genome was assembled into 582 scaffolds with a total size of 26.8 Mbp (Table S23 in Additional file 1). We also isolated sequences of bacterial origin from the BPH sequences and manually assembled the secondary bacterial symbiont genome, which is 90% identical to *Arsenophonus nasoniae* [50]. We tentatively designate this bacterium as *A. nilaparvatae*. We assembled this genome into 20 scaffolds and estimated a draft genome size of 2.96 Mbp (Table S23 in Additional file 1). These newly obtained draft genome sequences provide a first opportunity to study how BPH’s genome content complements those of its microbial endosymbionts under the monophagous diet of rice sap. Metabolic gene annotations revealed that the YLS and *A. nilaparvatae* provide complementary functions to the insect host in at least four aspects: essential amino acid synthesis, nitrogen storage and recycling, steroid synthesis, and vitamin supply.

**Figure 4:**
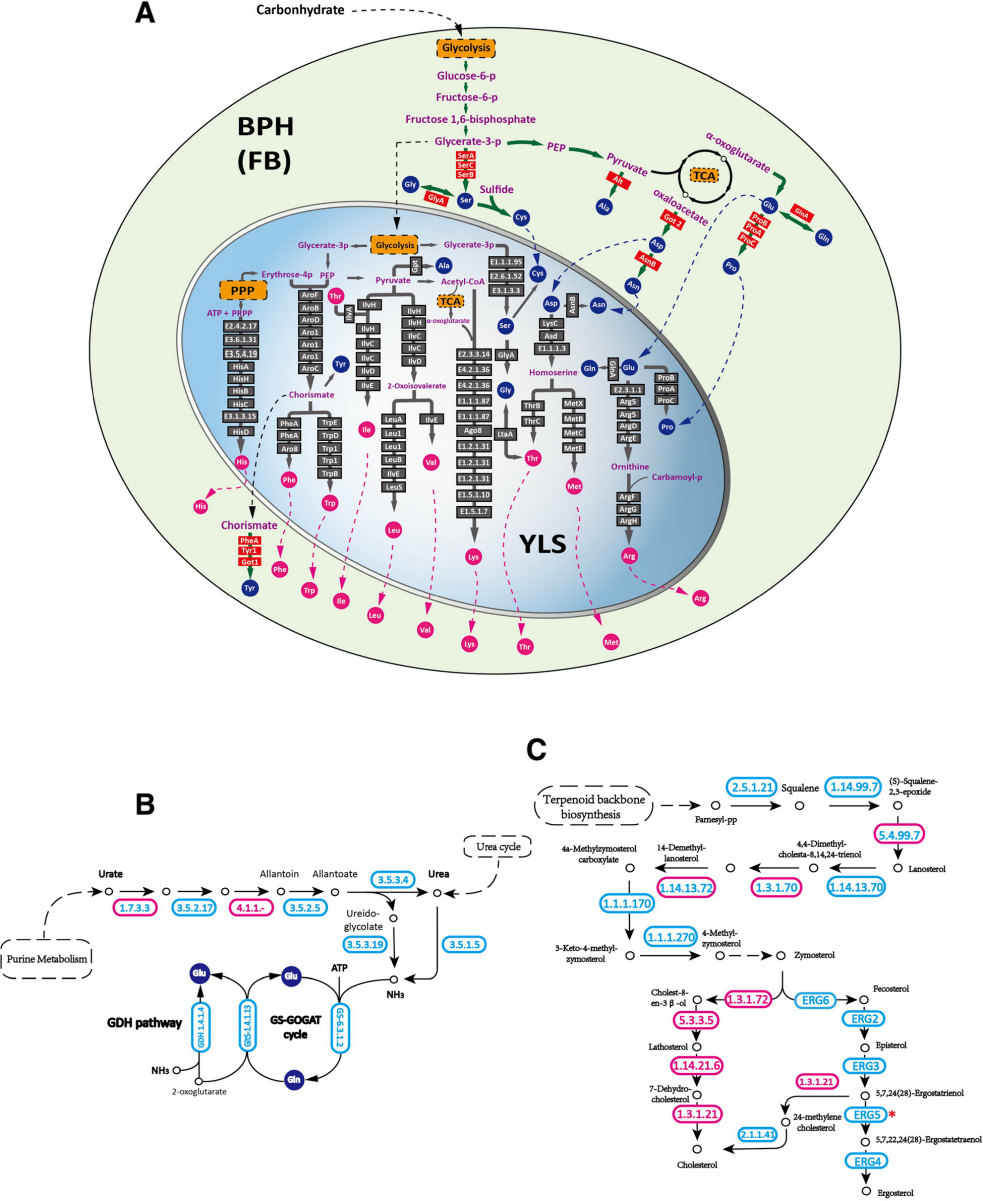
**Complementary metabolic pathways between the brown planthopper and its yeast-like symbiont. (A)** Interactions of the amino acid biosynthetic pathways of BPH and YLS within the fat body (FB). The green and blue areas represent the BPH fat body and endosymbiont cell, respectively. Essential amino acids are represented by solid pink circles and non-essential amino acids by solid blue circles. YLS genes are represented by grey boxes labeled with Enzyme Commission numbers or enzyme names corresponding to the Kyoto Encyclopedia of Genes and Genomes (KEGG) annotation of the YLS genome. BPH genes are represented by red boxes. **(B)** Genes involved in nitrogen recycling and ammonia assimilation pathways. **(C)** Genes involved in the steroid biosynthesis pathway. In (B,C), YLS genes are represented by blue ovals with blue numbers representing Enzyme Commission codes corresponding to the KEGG annotation of the genome. BPH genes are represented by pink ovals with pink numbers. Genes identified in both the YLS and BPH genomes are represented by pink ovals with blue numbers. A nonsense mutation was found in the *ERG5* gene (red asterisk).

First, annotation of metabolic genes indicated that YLS is able to provide essential amino acids that BPH is unable to synthesize (Figure 4A). We inferred from the BPH genome that, as expected, the insect host lacks the ability to carry out *de novo* synthesis of 10 essential amino acids (arginine, histidine, isoleucine, leucine, lysine, methionine, phenylalanine, threonine, tryptophan, and valine). Whereas most animals obtain essential amino acids from food, BPH’s sole food source - rice phloem sap - does not provide these necessary nutrients [51]. Thus, it requires an additional supply source. Our YLS genome sequence analysis indicates that this fungal symbiont has evolved a reduced genome size, yet it retains amino acid synthetic pathways that are highly complementary to the host, providing the first genomic evidence that all the genes required for essential amino acid biosynthesis exist in the YLS genome (Table S24 in Additional file 1), and this explains why BPH can survive on artificial diets that are depleted of these critical nutrients [52].

Second, we found that the genetic capacity for nitrogen recycling is complementary between BPH and YLS. Our analyses indicate that YLS has the genetic complement to allow it to use uric acid, a nitrogenous waste stored in the insect body, which is a novel mechanism of energy storage [47]. Annotation of genes from both the BPH and YLS genomes revealed complete pathways for nitrogen recycling and ammonia assimilation (Figure 4B; Table S25 in Additional file 1). The pathways of the two organisms are highly complementary, with only one gene shared between BPH and YLS (EC1.7.3.3; because of this minimal overlap, these pathways would be rendered non-functional if either BPH or its YLS lost just one of their current genes encoding the enzymes involved in them For example, we identified all the genes involved in urea recycling in the YLS genome except for the gene encoding the enzyme 2-oxo-4-hydroxy-4-carboxy-5-ureidoimidazoline decarboxylase (EC4.1.1.-), while this missing gene is present in the BPH genome (Figure 4B). In addition, BPH possesses uricase genes (EC1.7.3.3), which are rare in insects, including both hemipteran insects, based on currently available genome information. The retention or gain of these typically absent genes in BPH may be an evolutionary adaptation to facilitate nitrogen recycling by increasing urate production. This finding explains the observation that uricase activity can be detected in both whole insect tissues and in isolated YLS [47]. It also rejects the previous inference that planthoppers *per se* are devoid of uricase [53]. The close cooperation of the enzymes encoded by the two genomes serves to successfully transform urate into ammonia, which is further transformed into glutamate (Glu) and glutamine (Gln), the precursors for the biosynthesis of essential amino acids. This nitrogen-recycling pathway thereby serves as an additional source of amino acids for BPH, and indicates that this integrative system is an adaptation via host-symbiont co-evolution that enables BPH to exist solely on a diet of nutritionally limited and unbalanced rice phloem sap.

Third, we discovered that BPH and YLS have developed an interdependent system for steroid biosynthesis. Fungi commonly produce ergosterol, a component of yeast and fungal cell membranes that functions in a way similar to cholesterol in animal cells. We were able to identify all the genes involved in ergosterol synthesis in the YLS genome; however, our analysis confirms the presence of a previously reported nonsense mutation in the sterol C-22 desaturase gene *ERG5*, which catalyses ergosta-5,7,24(28)-trienol into ergosta-5,7,22,24(28)-tetraenol [54]. Thus, YLS is incapable of completing steroid synthesis and, as a consequence, ergosta-5,7,24(28)-trienol accumulates in it, and ergosterol production cannot be completed. Interestingly, we identified two genes in the BPH genome and YLS genome (encoding EC1.3.1.21 and EC2.1.1.41, respectively) that provide an alternative pathway, converting ergosta-5,7,24(28)-trienol into cholesterol through 24-methylenecholesterol [46]. Based on our findings, we further propose that BPH might utilize zymosterol, an intermediate generated by the YLS, to synthesize cholesterol (Figure 4C; Table S26 in Additional file 1). Again, the presence of YLS’s ability to supply sterol may explain how BPH can survive on a sterol-free diet [52].

The other bacterial symbiont genome in BPH that we sequenced is that of *A. nilaparvatae*, whose association with BPH serves an unknown role. *Arsenophonus* probably has an important role in BPH because BPH individuals that lack *Arsenophonus* always have *Wolbachia* [55], an endosymbiont bacterium commonly found in insects. Our analysis of the gene complement of all three associated genomes showed that both the YLS and BPH genomes had gene deficiencies in several vitamin biosynthesis pathways. However, we found that the *A. nilaparvatae* genome contained the complete functional gene set for B vitamin synthesis, suggesting it might supply B vitamins to BPH (Table S27 in Additional file 1). Further, the *Wolbachia* symbiont in the bed bug *Cimex lectularius* also provides B vitamins for its host [56], which supports the idea that *Arsenophonus* and *Wolbachia* play a similar role in their BPH host, and may potentially compete with each other. The capacity of *A. nilaparvatae* to supply vitamins to the host and the resultant co-evolution likely underly the reason for its widespread presence in BPH.

### Wing development in response to rice growth stages

One evolutionary adaptation that is thought to be essential to the success of BPH is its striking wing dimorphism, where the brachypterous (short-winged) females achieve proliferation potency on the rice plant and the macropterous (long-winged) females carry out long distance migration (Figure 1), allowing BPH to exploit its sole rice host over a range of regions when it becomes seasonally available. The wing dimorphism in BPH is a phenotypic plastic trait that is nutrition-inducible: rice at yellow maturity (low level nutrition) and tillering (high level nutrition) stages induce BPH nymphs to become macropterous-dominant or brachypterous-dominant adults, respectively [57,58], a crucial adaptation for BPH to find its exclusive host plant efficiently. In holometabolous insects, a series of signaling pathways were shown to jointly regulate the development of wing patterns [59,60]. We identified all wing development network genes in the BPH genome, which share a high level of sequence identity with genes in other sequenced insect genomes (Table S28 and Figure S20 in Additional file 1). Almost all of the identified genes had increased expression during the second to fifth instars of macropterous nymphs that were fed on yellow maturity rice plants (Figure S21a in Additional file 1). RNA interference of genes that had significantly increased expression in the third and fourth instars (for example, *ap* and *wingless*) interrupted wing development of both wing morphs (Figure S21b in Additional file 1), indicating these genes might be involved in the differentiation of wing development.

## Conclusion

Whole genome sequencing of BPH and its fungal and bacterial endosymbionts revealed genomic mechanisms of insect-symbiont interactions. The complementarity of the three genomes with regard to nutritional pathways, including essential amino acid and steroid biosynthesis by the fungal symbiont and vitamin B supplementation by the bacterial symbiont, enables BPH to thrive on a low-nutrient diet provided solely by rice. In addition, the contractions or loss of genes involved in chemoreception, detoxification, and digestion by BPH also reflect its unique sap diet. These three, complementary genomes will serve as invaluable resources for further understanding plant-herbivore-microbe interactions and the underlying evolutionary mechanisms involved in such mutualistic relationships. Genomic annotation and expression analysis indicated the wing network related to BPH’s dimorphism. All these findings highlight potential directions for effective pest control of BPH. Additionally, with a reference sequence available, more extensive re-sequencing in different global populations will improve the understanding of BPH’s migratory routes, and aid in identifying potential differences between populations that cause different levels of destruction.

## Materials and methods

### Strain selection and DNA preparation

The *N. lugens* population sequenced in the present study was originally collected in Hangzhou, China, in 2008 and had been reared on rice seedlings (strain: TN1). One male and one female were isolated and mated to produce F_1_ progeny. A single pair was then selected for sibling inbreeding for 13 generations. Genomic DNA was extracted from adult females of the F_13_ generation.

Female adults were collected and dissected under a Leica S8AP0 stereomicroscope (Leica, Germany) in order to remove the microbial symbionts. The insects were quickly washed in ice-cold phosphate-buffered saline (PBS) solution (137 mM NaCl, 2.68 mM KCl, 8.1 mM Na_2_HPO4, 1.47 mM KH_2_PO4 (pH 7.4)). Genomic DNA was then extracted from approximately 5,000 female *N. lugens* individuals using Wizard Genomic DNA Purification Kit (Promega, Madison, WI, USA) following the manufacturer’s protocols.

### Whole-genome shotgun sequencing and assembly

We constructed a series of DNA libraries with varying insert sizes (180 bp, 200 bp, 500 bp, 2 kb, 5 kb, 10 kb, 20 kb, and 40 kb) to perform WGS sequencing. For small insert size (<1 kb) libraries, we followed the manufacturer’s protocols (Illumina, Inc., San Diego, CA, USA). For large insert size (>1 kb) mate-paired libraries, approximately 20 to 50 μg of genomic DNA was fragmented, biotin labeled, self-ligated to form circularized DNA, merged at the ends of the DNA fragments, fragmented into linear DNA segments again, enriched using biotin/streptavidin, and then prepared for sequencing. All of the above libraries were sequenced on Illumina HiSeq 2000 or GA-II sequencers. In total, we constructed 16 DNA libraries, accounting for 21 Illumina HiSeq lanes. We generated 263.8 Gbp of raw WGS sequence data, covering approximately 219.81X of the BPH genome (Table S1 in Additional file 1). After data filtering and error correction, the short reads were assembled using SOAPdenovo [61].

### Data filtering and error correction

For both fosmid and conventional WGS sequence data, the raw reads generated from the Illumina pipeline included adapter-contaminated reads, low-quality reads, and duplicate reads. We filtered these reads to compile a clean dataset. For the fosmid sequence data, we also filtered out vector sequences.

Additionally, reads satisfying any of the following conditions were removed: (1) N constituting >2% of the bases for small insert size libraries or >10% of the bases for large insert size libraries; (2) contained poly-A sequences; (3) ≥40% of the bases possessing a low Phred quality value (<8) for small insert size libraries or ≥60% bases for large insert size libraries; (4) >10 bp aligned to the adapter sequence (allowing <4 bp mismatches); (5) an overlapping sequence length >10 bp (allowing 10% mismatches); or (6) duplicate reads that were identical paired reads generated by PCR.

Error correction was performed for the filtered small insert size library reads to eliminate sequencing errors prior to assembly. Low frequency k-mers with a cutoff value set at 10, which were defined by 17-mer analysis, were corrected to high frequency k-mers or trimmed at the end of the clean reads.

At last, a total of 158.01 Gbp of clean WGS data (131.67X of the genome) with 131.7-fold of genome coverage depth (Table S2 in Additional file 1) and were combined with 507.94 Gbp of clean fosmid sequence data for subsequent genome assembly and analysis.

### Evaluations of genome size and heterozygosity

Nuclear DNA content and the genome size of *N. lugens* were estimated first by flow cytometric analysis. A *N. lugens* female or male adult was ground in 1 ml PBS-T (PBS +0.5% TritonX-100) buffer using heads of adult *D. melanogaster* as an internal standard. The mixture was passed through a 30 μm CellTrics filter (Partec, Münster, North Rhine-Westphalia, Germany), treated with 2 μg/ml RNase A for 15 minutes at 37°C and stained with 5 μg/ml propidium iodide (PI) for 30 minutes at 25°C. Fifty-thousand cell nuclei in the suspension were analyzed on an EPICS Elite Analyzer (Beckman Coulter, Brea, California, USA) equipped with an argon laser emitting 15 mW at 488 nm. The major diploid peaks (2C) of propidium iodide fluorescent emissions were determined in the histogram generated by the EXPO32 software (Beckman Coulter) (Figure S1A in Additional file 1). The mean C value or genome size of *N. lugens* was calculated based on those of *D. melanogaster*, 0.18 pg or 175 Mbp. The process was replicated three times. Tissues from *Bombyx mori* and *Locusta migratoria* were also analyzed in the same experimental procedure in order to evaluate the reliability of the method.

We also examined the genome size and heterozygosity of the BPH genome using a k-mer approach. We used approximately 35 Gbp of the WGS sequencing data to generate a 17-mer depth-frequency curve. This preliminary result is shown in Figure S1B in Additional file 1, where two peaks can be observed. Subsequently, we used approximately 64 Gbp of the WGS sequencing data to generate a 27-mer depth-frequency curve (Figure S1C in Additional file 1). The first (left) peak observed in Figure S1B in Additional file 1 was elevated following increased sequence data (Figure S1C in Additional file 1). Thus, it was considered to be the heterozygosis peak. Therefore, we concluded that the main peak occurred at 24, and the total input read number is 355,220,940. Given that the total base number is 34,969,752,790 and the total k-mer number is 29,286,217,750 (Table S3 in Additional file 1), we calculated the genome size of BPH to be 1.2 Gbp based on the following formula: Genome size = k-mer_num/Peak_depth [61].

### Fosmid library construction, sequencing and assembly

To overcome the challenge caused by high heterozygosity and repeat sequence content, we employed a recently developed genome sequencing strategy that combines WGS with pooled fosmid sequencing [62]. We constructed 48,096 fosmid clones and plasmid DNA extracted from each fosmid clone was tagged with a unique index and adapter and then sheared to construct two small insert size libraries (250 bp and 500 bp). DNA from every approximately 2,016 fosmid clones was pooled for sequencing for both the 250 bp and 500 bp libraries. In total, we generated 925.6 Gbp of raw data (46 Illumina HiSeq lanes) from the fosmids. After data filtering and error correction, we obtained a total of 507.9 Gbp of clean fosmid data (Table S4 in Additional file 1).

Error-corrected clean data for the short insert size libraries of all fosmids were assembled using SOAPdenovo [61], followed by gap filling. The assembly procedures included splitting reads into k-mers, constructing a de Bruijn graph, producing contig sequences, realigning reads to contig sequences to construct scaffolds, and filling gaps within scaffolds using GapCloser [61]. These fosmid clones provided approximately 1.6-fold genome coverage.

Each of the fosmid libraries contained one of the two allelic sequences of a small proportion of the genome (40 kbp). A series of randomly selected haploid fosmid sequences were pooled, shotgun sequenced and assembled. This technical improvement was made based on the individual fosmid-sequencing pipeline described in a study of the diamond-back moth genome [13] and significantly reduced the overall cost of individual library construction.

### Genome assembly

We combined the fosmid and WGS assembly results using Rabbit [63]. The assembly procedure is shown in Figure S4 of Additional file 1 and explained in detail in You *et al*. [13]. The assembly pipeline that deals with the heterozygosity issue is shown in Figure S5 in Additional file 1. Briefly, a diploid genome consists of regions containing only unique genes (sequences that occur only once in a haploid genome) and regions containing repetitive sequences (including high-repeat repetitive components and homologous genes), as shown in Figure S5A,B in Additional file 1. Potentially, heterozygous alleles could be assembled into two copies of the same gene and mistakenly presented as homologs on the same haploid genome. Therefore, we employed a procedure to distinguish heterozygous sequences from duplications and to identify erroneous assemblies caused by hyterozygosity. The Rabbit software only merges sequences with similarity >95% and maintains all sequences with low similarity in the library (Figure S5A in Additional file 1). Heterozygous sequences could be distinguished from real gene duplications by examining the frequency and distribution of k-mers using WGS reads (Figure S5B in Additional file 1) [13]. If a unique sequence (identified by k-mer analysis) was represented in two sequences from the assembly, we considered them as heterozygous sequences and removed the shorter copy (Figure S5C in Additional file 1). Thus, this procedure eliminates the majority of heterozygous sequences from the final assembly. Finally, we produced an assembly of 1.141Gbp with a scaffold N50 of 356,597 bp (Table S5 in Additional file 1).

### Distribution of sequence depths

We aligned filtered reads (clean WGS data) onto the assembled genome using *SOAP* and calculated the percentages of base pairs for each given sequencing depth along the genome. The results showed that the percentage of bases with less than a five-read coverage was only approximately 2% (Figure S3 in Additional file 1), indicating that the genome had been extensively covered by sequencing reads.

### EST evaluation of genome coverage for gene regions

Transcript sequences were used as queries and mapped to the assembled genome sequences to examine the coverage rate for these EST data generated by transcriptome assembly. Overall, About 96% of the ESTs are covered by our genome assembly, with >50% of these regions found in one complete scaffold (Table S6 in Additional file 1), indicating that the majority of protein-coding genes had been successfully assembled in our genome.

### CEGMA evaluation

We evaluated the coverage rate of the BPH_v1 genome assembly against 248 core eukaryotic genes using the CEGMA 2.4 [14] method and compared our results with those for the *A. pisum* genome (Tables S7 and S8 in Additional file 1).

### GC content

We used 50-kbp non-overlapping sliding windows and calculated the GC content and average depths among these windows. Each window represents 50 kbp of genome sequence. For each of these windows, the average depth was calculated by mapping all clean WGS reads to the genome using *SOAPaligner*. The sequencing depth for each base was counted, and the average depth was computed from all bases in the window (Figure S7 in Additional file 1).

### Repetitive element annotation

We identified TEs from the BPH genome using two methods: searching for homologous repeats against the Repbase [64] TE library using RepeatMasker [65] and RepeatProteinMask [65]; and constructing a *de novo* repeat library using repeatmodeler [66], followed by finding TE repeats using RepeatMasker. In detail, first we identified known TEs by homolog-based annotation in the *N. lugens* genome using RepeatMasker and RepeatProteinMask, which are based on the Repbase database. Second, an *ab initio* repeat library, which combined repeatmodeler and LTR-FINDER-1.0.5, was used as a custom library in RepeatMasker to identify homologs in the genome and to classify the found repeats. Finally, we eliminated the overlap between the two methods and obtained the combinational data. The tandem repeats were annotated using the software Tandem Repeats Finder [67].

The divergence rates were calculated for BPH TEs identified by the two different approaches (Figure S6a,b in Additional file 1). The divergence rate was calculated for each TE repeat as the percentage of mismatches compared with the consensus sequence in the Repase/Denovo library. Comparison of the distributions of TEs predicted by these different methods indicated that lineage-specific TEs (especially DNA transposons) identified by the *de novo* approach had evolved relatively recently. These findings suggest that recent TE activity may have contributed to the formation of the large genome of BPH.

### Gene annotation

Gene structure prediction was performed using three methods: homology-based prediction, *de novo* prediction, and RNA-seq assisted prediction. Protein sequences from five species (*A. pisum*, *T. castaneum*, *A. mellifera*, *P. humanus*, and *Homo sapiens*) were used as query sequences and mapped to the genome using TblastN with an E <1e-5. We then predicted segmented exon-intron gene structures using GeneWise [17] by aligning query proteins to mapped genome fragments. Meanwhile, we used AUGUSTU*S* [19] and GENSCAN [18] to predict gene models on the repeat-masked genome, where short genes with only one or two exons were filtered out. We combined the homology-based and *de novo* prediction gene models with GLEAN [68]. Additionally, we aligned transcriptome reads against the genome using TopHat [69] and predicted gene models with Cufflinks [70]. A final gene set was generated by integrating RNA-seq predicted gene models and the GLEAN gene set.

Annotation of gene function was performed by aligning protein sequences to both the SwissProt [71] and TrEMBL [71] databases. We annotated motifs and domains using InterProSca*n* [72] against publicly available databases including ProDom, PRINTS, Pfam, SMART, PANTHER, and PROSITE. The description of gene products was performed by gene ontology [73], which was retrieved from the results of InterProScan. We also aligned annotated genes to the Kyoto Encyclopedia of Genes and Genomes (KEGG) [74] and constructed the corresponding KEGG pathway map.

### Predicted genes mapped with transcriptome sequences and RT-PCR validation

To test whether the predicted genes are actively transcribed, we used a unigene set derived from *de novo* assembly of transcriptomes to map the 27,571 predicted genes. We then calculated the proportion of annotated and unannotated genes mapped with transcripts (Table S9 in Additional file 1). To further validate the expression of *N. lugens*-specific genes, we randomly chose 30 from the unannotated genes with RNA sequence support (Table S9 in Additional file 1) for RT-PCR and sequencing (Figure S8 and Table S10 in Additional file 1).

### ncRNA annotation

Four types of ncRNAs were annotated in our analysis. They are miRNA, tRNA, rRNA, and snRNA. We detected four types of non-coding RNAs in the genome by performing homologous searching across the whole-genome sequence. tRNAscan-SE (v.1.23) [75] was used for tRNA prediction. snRNAs and miRNAs were predicted by alignment using BlastN and searching with INFERNAL (v.0.81) against the Rfam database [76]. rRNAs were found by BlastN alignment against human rRNA as reference sequences.

### Ortholog prediction

We included all major insect lineages with genome sequences publically available, including a third hemipteran species, *R. prolixus*, obtained from [77], for ortholog prediction and phylogenetic reconstruction. We identified gene families using TreeFam [78,79] following these steps: 1) BLASTP was used to compare all protein sequences for the 15 selected species, including BPH, with an E-value <1e^-7^; 2) The High-scoring Segment Pair (HSP) segments between the same pair of proteins were concatenated using Solar[79], which was followed by identification of homology among protein sequences based on the bit-scores and identities of homologous gene pairs; and 3) gene families were identified by clustering using Hcluster_sg (v.0.5.0, build 4 April 2007) [79], whose algorithm was similar to that of regular hierarchical clustering.

### Phylogenetic reconstruction and divergence time estimation

Single-copy gene families were used to reconstruct the phylogenetic relationships. Coding sequences from each single-copy family were concatenated to form one super gene for each species. All of the nucleotides at codon position 2 of these concatenated genes were extracted and used to construct the phylogenetic tree using PhyML [80,81], with a gamma distribution across sites and an HKY85 substitution model.

The same set of sequences at codon position 2 was used to estimate divergence times among lineages. Fossil calibrations were set according to previous papers [82,83]. The PAML mcmctree (v.4.5) [84-86] program was used to compute split times using the approximate likelihood calculation algorithm. Tracer (v.1.5.0) [87] was applied to examine convergence, and two independent runs were performed for confirmation.

### Evaluation of chemoreception gene assembly

RNA was extracted from 20 individuals of *N. lugens* using TRIzol reagent (Invitrogen, CA, USA) according to the manufacturer’s protocol. Extracted RNA was treated with DNase (Takara, Japan) to remove any genomic DNA contaminant and was reverse transcribed to produce cDNA using PrimeScript 1^st^ strand cDNA synthesis kit (Takara). Gene-specific primers were designed (Table S29 in Additional file 1) and synthesized for the cDNAs of randomly selected chemoreception system genes. The annealing temperature was determined for PCR amplification of each cDNAs. Quality of PCR products was evaluated by electrophoresis on agarose gels. DNA bands of the expected size were excised from the agarose gel and purified using DNA gel extraction kit (Axygen, Union City, California, USA). These PCR products were cloned into pMD18-T vector (Takara). At least three independent clones were Sanger sequenced from each cDNA (Major Biotech., Shanghai, China).

To evaluate the quality of *de novo* assembly of the chemoreception genes, nucleotide sequence data obtained via searching the assembled BPH genome and open reading frame (ORF) prediction were compared against the local *N. lugens* transcriptome database using BLASTN [88]. As a result, all OBP and CSP gene sequences obtained from transcriptome data were matched to their counterparts from the genomic prediction, whereas the number of genes found from the genomic prediction was more than twice that in the transcriptome data. Moreover, six OR and GR gene sequences assembled in the genome did not seem to have any counterparts in the transcriptome, which was likely a consequence of their low expression levels in the transcripts. Thus, it is essential to use the genome-based assembly approach to identify these genes.

### Evolutionary reconstruction of chemoreception gene families

Chemoreception genes of a number of insects were obtained from GenBank. For evolutionary reconstruction of OBP genes, genes from 13 insect species, including *A. mellifera*, *B. mori*, *D. melanogaster*, *A. pisum*, *T. castaneum*, *Brevicoryne brassicae*, *Adelphocoris lineolatus*, *Euschistus heros*, *Sitobion avenae*, *Apolygus lucorum*, *Myzus persicae*, *Drepanosiphum platanoidis*, and *N. lugens*, were selected. For evolutionary reconstruction of CSP genes, genes from 12 insect species were selected: *A. mellifera*, *B. mori*, *D. melanogaster*, *A. pisum*, *T. castaneum*, *Anopheles gambiae*, *A. lineolatus*, *E. heros*, *S. avenae*, *M. persicae*, *Lipaphis erysimi*, and *N. lugens*. For evolutionary reconstruction of OR genes, genes from six insect species were selected: *A. mellifera*, *B. mori*, *D. melanogaster*, *A. pisum*, *Heliothis virescens*, and *N. lugens*. For evolutionary reconstruction of GR genes, genes from three insect species were selected: *D. melanogaster*, *A. pisum*, and *N. lugens*.

### Reconstruction for phylogenetic histories of chemoreception genes

Phylogenetic tree inferences were made using RAxML v.7.28 [89] by applying a general time reversible (GTR) model of nucleotide substitution with a gamma model of rate heterogeneity using the JTT matrix [90] and bootstrapping for 100 times. Phylogenetic trees were then presented in circular shape and colored taxonomically using online tools provided by iTOL [91].

The phylogeny of GR genes indicates that some BPH GRs may be involved in detection of sugars. For example, *NlGR10* is clustered with *DmGr43a*, which is a narrowly tuned fructose receptor in taste neurons and in the brain, and functions as a nutrient sensor for hemolymph fructose and assigns opposing valence to feeding experiences in a satiation-dependent manner [92]; *NlGR5*, *NlGR1* and *NlGR2* are all clustered with the sugar receptor genes *DmGr5a* and *DmGr64a* and other putative sugar receptor genes from other insects [93]. BPH GRs may also be functional in odor detection: *NlGR6* is clustered with *DmGR10a*, which encodes a protein whose best known ligand is ethylbenzoate and may contribute to the detection of odors along with *DmOr10a* instead of gustatory function [94]. *NlGR3*, *NlGR4* and *NlGR7* are clustered with the GR gene from *A. pisum*, and are close to the well-known CO_2_ sensor genes *DmGr21* and *DmGr64* [95].

### Differential gene expression in various tissues

For studies of tissue-specific expression of selected chemoreception genes, a series of adult *N. lugens* were dissected in ice-cold RNAlater (Ambion, Austin, TX, USA). Two replicates for four tissue types - antennae, remaining heads (that is, head without antennae), legs, and remaining bodies (that is, body without heads and legs) were prepared, each containing tissues from >300 fresh adults. Tissues were then stored in RNAlater at −80°C until RNA extractions were carried out. RNA was extracted using TRIzol reagent (Invitrogen, CA, USA) according to the manufacturer’s protocol.

### Quantitative real-time PCR assay

The concentration of each RNA sample was adjusted to 1 μg/μl with nuclease-free water before it was reverse transcribed in a 20 μl reaction system using the AMV RNA PCR Kit (Takara). The quantitative RT-PCR was performed on an ABI 7300 Real-Time PCR System (Applied Biosystems, Branchburg, NJ, USA) using the SYBR Premix Ex Taq Kit (Takara) with primers developed in this study, according to the manufacturer’s protocol. A negative control (nuclease-free water) was included through the experiments to detect contamination and to determine the degree of dimer formation. After the quantitative RT-PCR assay, the results (threshold cycle values) were normalized to the expression level of the constitutive alpha-tubulin gene, which was obtained *a priori* for each tissue replicate via pre-run experiments. A relative quantitative method (^ΔΔ^Ct) was used to evaluate the expression variation among tissues [96].

### Genomes of yeast-like symbionts

The YLSs were isolated from their BPH host following the method by Noda and Omura [97] (Figure S14 in Additional file 1). Five grams of BPH were homogenized in 0.85% NaCl solution. The homogenate was filtered through cotton cloth and centrifuged for 5 minutes at 100 *g*. The pellet was resuspended twice. The suspension of the final pellet, mixed with 4 volumes of Percoll (GE Healthcare, Uppsala, Sweden) containing 0.25 M sucrose, was centrifuged for 30 minutes at 82,000 *g* in a Hitachi ultracentrifuge (Himac CP80MX) using P40ST horizonal rotor. Genomic DNA was extracted using the Yeast Smash & Grab DNA miniprep method described by Rose *et al*. [98]. Isolated YLS was dissolved in 200 μl of lysis buffer (100 mM NaCl, 1 mM EDTA (pH 8.0), and 10 mM Tris-HCl (pH 8.0), 1% SDS, 2% Triton X-100), and treated with lyticase (Sigma, St. Louis, Missouri, USA) at 37°C for 30 minutes, then mixed with the same volume of phenol/chloroform (1:1), and vortexed vigorously with glass beads (425 to 600 μm; Sigma). Aqueous phase was recovered by ethanol precipitation and the pellet was redissolved in Tris EDTA buffer (10 mM Tris, bring to pH 8.0 with HCl, 1 mM EDTA).

The genome of YLS was shotgun sequenced on a Roche 454 GS FLX at the Chinese National Human Genome Center at Shanghai. A total of 573,847 reads were generated and used to construct contigs using the Newbler software [99] with default parameters. Additionally, DNA libraries of 300 bp and 3 kb insert sizes were constructed and sequenced using an Illumina Hiseq 2000. A total of 10,550,032 Illumina paired-end reads and 7,731,568 mate-pair reads were produced and used for scaffolding using the SSPACE software with parameters: -m 30 -o 20 -k 5 -a 0.7 -× 1[100]. A total of 28.4 Mbp genome sequences were assembled. In addition, sequencing of genomic fosmid libraries of a whole female BPH body generated 20.9 Mbp YLS Illumina sequences. Finally, with all data combined, the YLS genome was assembled into 26.8 Mbp.

### Genome of the bacterial symbiont

As part of the BPH genome project, a total of 9.3 Mbp putative bacteria genome sequences combined with 5.7 Mbp from BPH fosmid library data were filtered from BPH genome sequence data. To ensure the quality of the assembled BPH genome, several steps were carried out for filtering contaminated sequences. According to different characteristics of GC content distribution in different species genomes, WGS data were mapped to the genome to plot a GC depth distribution diagram. The scaffolds located in a patch of separated region were filtered out and submitted to blast against the nt database with a cutoff E-value of 1e-5. As a result, these scaffolds were homologous to some bacterial species, such as *Escherichia coli*, and were discarded. The assembled scaffolds of the *N. lugens* genome were further filtered during data submission to GenBank to eliminate any remaining bacterial sequence contaminates (accession numbers AOSB00000000; BioProject PRJNA177647). The final assembled version submitted to GenBank was used for analysis in this manuscript. Then we used the *A. nasoniae* protein sequences as the reference data, which are available from the NCBI with project accession PRJEA37749, to search against the assembled BPH genome with a cutoff E-value of 1e-10. Finally, the homologous sequences obtained were manually assembled into the bacterial genome. Additional contaminated sequences were filtered during manual assembly to ensure the quality of the bacterial genome. The assembly is very high quality, with a total scaffold number of 20 with an N50 of 199,718 bp. To further confirm the assembled bacterial scaffolds, we searched the generated sequences against the reference database using algorithm BLASTX with a cutoff E-value of 10^-10^.

### Gene annotation of YLS and *A. nilaparvatae* and functional pathway analysis

Annotations of the genomic sequence of YLS was performed with *AUGUSTUS* and the annotated information of *Metarhizium acridum* was incorporated as a reference [101]. Meanwhile, GLIMMER3.0 was used to predict genes of *A. nilaparvatae*. Annotation of gene function was performed by aligning protein sequences to both the SwissProt and TrEMBL databases. Then we aligned annotated genes to KEGG databases and constructed the corresponding KEGG pathway map.

### Wing development and RNA interference

Genes relevant to wing patterns known from previous studies in aphid and beetle [22,63] were identified for BPH in the present study. We found wing network genes in the BPH genome using BLAST. Some of these wing network genes exhibited duplications. Many of these genes were involved in anterior-posterior, dorsal-ventral, and body-wall/wing development.

For RNA interference, total RNA was isolated from *N. lugens* samples by using the TRIzol Total RNA Isolation kit (Takara). First-strand cDNA was synthesized by the First Strand cDNA Synthesis kit (TIANGEN, China) using an oligo(dT)_18_ primer and 1 μg total RNA template in a 20 μl reaction volume following the manufacturer’s protocol. All target genes were cloned and confirmed by sequencing. Double-stranded RNAs were synthesized from linearized templates prepared by RT-PCR amplification using MEGAscript T7 Transcription kit (Ambion, Austin, TX, USA) and gene-specific primers (Table S29 in Additional file 1). Unique regions of each *N. lugens* target gene were chosen as templates for synthesizing gene-specific double-stranded RNA. The third and fourth instar nymphs were used for microinjection, where 30 nymphs were used in each treatment for each of the three replicates. Double-stranded RNA preparation and detailed microinjection procedures followed those of Wang *et al.* [102]. Six insects were randomly selected for treatment and control groups at the second and third days after injection, and the total RNA was extracted to calculate the relative expression level by RT-PCR and quantitative PCR.

### Data availability

The BPH genome assemblies have been deposited at GenBank under accession number AOSB00000000 (BioProject PRJNA177647). The YLS and *Arsenophonus* endosymbiont are at GenBank under accession numbers JRMI00000000 and JRLH00000000, respectively. The BPH and *Arsenophonus* endosymbiont genome raw data have been deposited in the Sequence Read Archive (SRA) database [103] under accession number SRA183062. The YLS genome raw data are in the SRA database under accession number SRP048633. The transcriptomic sequences are in the SRA database with accession number SRX023419.

## Additional file

Additional file 1:
**Supplementary Figures S1 to S21 and Tables S1 to S30.**

## Abbreviations

bp: base pair
BPH: brown planthopper
CDS: coding sequence
CSP: chemosensory protein
EST: expressed sequence tag
GR: gustatory receptor
GST: glutathione-S-transferase
KEGG: Kyoto Encyclopedia of Genes and Genomes
miRNA: microRNA
ncRNA: non-coding RNA
OBP: odorant binding protein
OR: odorant receptor
PBS: phosphate-buffered saline
PCR: polymerase chain reaction
PM: peritrophic matrix
snRNA: small nuclear RNA
TE: transposable element
WGS: whole-genome shotgun
YLS: yeast-like symbiont

## References

[1] Zeigler RS, Heong KL, Hardy B (2009). Preface. Planthoppers: New Threats to the Sustainability of Intensive Rice Production Systems in Asia.

[2] Cheng JA, Heong KL, Hardy B (2009). Rice Planthopper Problems and Relevant Causes in China. Planthoppers: New Threats to the Sustainability of Intensive Rice Production Systems in Asia.

[3] Bottrell DG, Schoenly KG (2012). Resurrecting the ghost of green revolutions past: the brown planthopper as a recurring threat to high-yielding rice production in tropical Asia. J Asia Pac Entomol.

[4] Horgan F, HK L, Hardy B (2009). Mechanisms of Resistance: A Major gap in Understanding Planthopper-Rice Interactions. Planthoppers: New Threats to the Sustainability of Intensive Rice Production Systems in Asia.

[5] Sogawa K (1982). The Rice Brown Planthopper - Feeding Physiology and Host Plant Interactions. Annu Rev Entomol.

[6] Cook A, Denno RF, Denno RF, Perfect TJ (1994). Planthopper/Plant Interactions: Feeding Behavior, Plant Nutrition, Plant Defense, and Host Plant Specialization. Planthoppers: Their Ecology and Management.

[7] Lu ZX, Yu XP, Chen JM, Zheng XS, Xu HX, Zhang JF, Chen LZ (2004). Dynamics of yeast-like symbiote and its relationship with the virulence of brown planthopper, *Nilaparvata lugens* Stål, to resistant rice varieties. J Asia Pac Entomol.

[8] Chen YH, Bernal CC, Tan J, Horgan FG, Fitzgerald MA (2011). Planthopper ‘adaptation’ to resistant rice varieties: Changes in amino acid composition over time. J Insect Physiol.

[9] Cheng SN, Chen JC, Hsue S, Yan LM, Chu TL, Wu CT, Chien JK, Yan CS (1979). Studies on the migrations of brown planthopper *Nilaparvata-Lugens* Stal. Acta Entomol Sin.

[10] Perfect TJ, Cook AG, Denno RF, Perfect TJ (1994). Rice planthopper dynamics: a comparison between temperate and tropical regions. Planthoppers: Their Ecology and Management.

[11] Riley JR, Cheng XN, Zhang XX, Reynolds DR, Xu GM, Smith AD, Cheng JY, Bao AD, Zhai BP (1991). The long-distance migration of *Nilaparvata-Lugens* (Stal) (Delphacidae) in China - radar observations of mass return flight in the autumn. Ecol Entomol.

[12] Denno RF: **Life History Variation in Planthoppers.** In *Planthoppers.* Springer; 1994:163–215.

[13] You M, Yue Z, He W, Yang X, Yang G, Xie M, Zhan D, Baxter SW, Vasseur L, Gurr GM, Douglas CJ, Bai JL, Wang P, Cui K, Huang SG, Li XC, Zhou Q, Wu ZY, Chen QL, Liu CH, Wang B, Li XJ, Xu XF, Lu CX, Hu M, Davey JW, Smith SM, Chen MS, Xia XF, Tang WQ (2013). A heterozygous moth genome provides insights into herbivory and detoxification. Nat Genet.

[14] Parra G, Bradnam K, Ning Z, Keane T, Korf I (2009). Assessing the gene space in draft genomes. Nucleic Acids Res.

[15] The International Aphid Genomics C (2010). Genome sequence of the pea aphid *Acyrthosiphon pisum*. PLoS Biol.

[16] Adams MD, Celniker SE, Holt RA, Evans CA, Gocayne JD, Amanatides PG, Scherer SE, Li PW, Hoskins RA, Galle RF, George RA, Lewis SE, Richards S, Ashburner M, Henderson SN, Sutton GG, Wortman JR, Yandell MD, Zhang Q, Chen LX, Brandon RC, Rogers YH, Blazej RG, Champe M, Pfeiffer BD, Wan KH, Doyle C, Baxter EG, Helt G, Nelson CR (2000). The genome sequence of *Drosophila melanogaster*. Science.

[17] Birney E, Clamp M, Durbin R (2004). GeneWise and Genomewise. Genome Res.

[18] Burge C, Karlin S (1997). Prediction of complete gene structures in human genomic DNA. J Mol Biol.

[19] Stanke M, Keller O, Gunduz I, Hayes A, Waack S, Morgenstern B (2006). AUGUSTUS: ab initio prediction of alternative transcripts. Nucleic Acids Res.

[20] Elsik CG, Mackey AJ, Reese JT, Milshina NV, Roos DS, Weinstock GM (2007). Creating a honey bee consensus gene set. Genome Biol.

[21] Colbourne JK, Pfrender ME, Gilbert D, Thomas WK, Tucker A, Oakley TH, Tokishita S, Aerts A, Arnold GJ, Basu MK, Bauer DJ, Cáceres CE, Carmel L, Casola C, Choi J-H, Detter JC, Dong Q, Dusheyko S, Eads BD, Fröhlich T, Geiler-Samerotte KA, Gerlach D, Hatcher P, Jogdeo S, Krijgsveld J, Kriventseva EV, Kültz D, Laforsch C, Lindquist E, Lopez J (2011). The ecoresponsive genome of Daphnia pulex. Science.

[22] Richards S, Gibbs RA, Weinstock GM, Brown SJ, Denell R, Beeman RW, Gibbs R, Bucher G, Friedrich M, Grimmelikhuijzen CJ, Klingler M, Lorenzen M, Roth S, Schroder R, Tautz D, Zdobnov EM, Muzny D, Attaway T, Bell S, Buhay CJ, Chandrabose MN, Chavez D, Clerk-Blankenburg KP, Cree A, Dao M, Davis C, Chacko J, Dinh H, Dugan-Rocha S, Fowler G (2008). The genome of the model beetle and pest *Tribolium castaneum*. Nature.

[23] Consortium THGS (2006). Insights into social insects from the genome of the honeybee *Apis mellifera*. Nature.

[24] Campbell BC, Steffen-Campbell JD, Sorensen JT, Gill RJ (1995). Paraphyly of Homoptera and Auchenorrhyncha inferred from 18S rDNA nucleotide sequences. Syst Entomol.

[25] Smith BH, Getz WM (1994). Nonpheromonal olfactory processing in insects. Annu Rev Entomol.

[26] Visser JH, Piron PGM, Hardie J (1996). The aphids’ peripheral perception of plant volatiles. Entomol Exp Appl.

[27] Youn YN (2002). Electroantennogram responses of *Nilaparvata lugens* (Homoptera: Delphacidae) to plant volatile compounds. J Econ Entomol.

[28] Chenier JVR, Philogene BJR (1989). Field responses of certain forest Coleoptera to conifer monoterpenes and ethanol. J Chem Ecol.

[29] He P, Zhang J, Liu NY, Zhang YN, Yang K, Dong SL (2011). Distinct expression profiles and different functions of odorant binding proteins in *Nilaparvata lugens* Stal. PLoS One.

[30] McBride CS (2007). Rapid evolution of smell and taste receptor genes during host specialization in *Drosophila sechellia*. Proc Natl Acad Sci U S A.

[31] Li X, Schuler MA, Berenbaum MR (2007). Molecular mechanisms of metabolic resistance to synthetic and natural xenobiotics. Annu Rev Entomol.

[32] Karatolos N, Pauchet Y, Wilkinson P, Chauhan R, Denholm I, Gorman K, Nelson D, Bass C, Williamson M (2011). Pyrosequencing the transcriptome of the greenhouse whitefly, Trialeurodes vaporariorum reveals multiple transcripts encoding insecticide targets and detoxifying enzymes. BMC Genomics.

[33] Claudianos C, Ranson H, Johnson R, Biswas S, Schuler M, Berenbaum M, Feyereisen R, Oakeshott J (2006). A deficit of detoxification enzymes: pesticide sensitivity and environmental response in the honeybee. Insect Mol Biol.

[34] **AphidBase.** [http://www.aphidbase.com//]

[35] 35.**Flybase.** [ftp://ftp.flybase.org/genomes/Drosophila_melanogaster/dmel_r5.27_FB2010_04/]

[36] **Hymenoptera Genome database.** [http://www.hymenopteragenome.org/drupal/sites/hymenopteragenome.org.beebase/files/data/]

[37] **Vectorbase.** [ftp://ftp.vectorbase.org/public_data/organism_data/aaegypti/Geneset/]

[38] **Tribolium_castaneum.** [ftp://ftp.ncbi.nih.gov/genomes/Tribolium_castaneum]

[39] **Silkdb.** [ftp://silkdb.org/pub/release_2.0/]

[40] **VectorBase.** [http://www.vectorbase.org/GetData/Downloads/]

[41] **NasoniaBase.** [http://www.hymenopteragenome.org/nasonia/]

[42] Baumann P (2005). Biology bacteriocyte-associated endosymbionts of plant sap-sucking insects. Annu Rev Microbiol.

[43] Buchner P (1965). Endosymbiosis of Animals with Plant Microorganisms.

[44] Suh SO, Noda H, Blackwell M (2001). Insect symbiosis: derivation of yeast-like endosymbionts within an entomopathogenic filamentous lineage. Mol Biol Evol.

[45] Chen CC, Cheng LL, Kuan CC, Hou RF (1981). Studies on the intracellular yeast-like symbiote in the brown planthopper, *Nilapavarta-lugens* stal.1. histological observations and population-changes of the symbiote. J Appl Entomol.

[46] Wetzel JM, Ohnishi M, Fujita T, Nakanishi K, Naya Y, Noda H, Sugiura M (1992). Diversity in steroidogenesis of symbiotic microorganisms from planthoppers. J Chem Ecol.

[47] Hongoh Y, Ishikawa H (1997). Uric acid as a nitrogen resource for the brown planthopper, *Nilaparvata lugens*: Studies with synthetic diets and aposymbiotic insects. Zool Sci.

[48] Wan PJ, Yang L, Wang WX, Fan JM, Fu Q, Li GQ (2014). Constructing the major biosynthesis pathways for amino acids in the brown planthopper, Nilaparvata lugens Stål (Hemiptera: Delphacidae), based on the transcriptome data. Insect Mol Biol.

[49] Wang WX, Luo J, Lai FX, Fu Q (2010). Identification and phylogenetic analysis of symbiotic bacteria Arsenophonus from the rice brown planthopper, *Nilaparvata lugens* (Stal)(Homoptera: Delphacidae). Acta Entomol Sin.

[50] Wilkes TE, Darby AC, Choi JH, Colbourne JK, Werren JH, Hurst GD (2010). The draft genome sequence of *Arsenophonus nasoniae*, son-killer bacterium of *Nasonia vitripennis*, reveals genes associated with virulence and symbiosis. Insect Mol Biol.

[51] Hayashi H, Chino M (1990). Chemical composition of phloem sap from the uppermost internode of the rice plant. Plant Cell Physiol.

[52] Koyama K (1985). Nutritional physiology of the brown rice planthopper, *Nilaparvata lugens* Stal (Hemiptera: Delphacidae). II. Essential amino acids for nymphal development. Appl Entomol Zool.

[53] Hongoh Y, Sasaki T, Ishikawa H (2000). Cloning sequence analysis and expression in *Escherichia coli* of the gene encoding a uricase from the yeast-like symbiont of the brown planthopper, *Nilaparvata lugens*. Insect Biochem Mol Biol.

[54] Noda H, Koizumi Y (2003). Sterol biosynthesis by symbiotes: cytochrome P450 sterol C-22 desaturase genes from yeastlike symbiotes of rice planthoppers and anobiid beetles. Insect Biochem Mol Biol.

[55] Qu LY, Lou YH, Fan HW, Ye YX, Huang HJ, Hu MQ, Zhu YN, Zhang CX (2013). Two endosymbiotic bacteria, Wolbachia and Arsenophonus, in the brown planthopper Nilaparvata lugens. Symbiosis.

[56] Hosokawa T, Koga R, Kikuchi Y, Meng XY, Fukatsu T (2010). Wolbachia as a bacteriocyte-associated nutritional mutualist. Proc Natl Acad Sci U S A.

[57] Zhang ZQ (1983). A study on the development of wing dimorphism in the rice brown planthopper. Acta Entomol Sin.

[58] Hu DB, Luo BQ, Li J, Han Y, Jiang TR, Liu J, Wu G, Hua HX, Xiong YF, Li JS (2013). Genome-wide analysis of Nilaparvata lugens nymphal responses to high-density and low-quality rice hosts. Insect Sci.

[59] Zhan S, Merlin C, Boore JL, Reppert SM (2011). The monarch butterfly genome yields insights into long-distance migration. Cell.

[60] Gould JL (1998). Sensory bases of navigation. Curr Biol.

[61] Li R, Fan W, Tian G, Zhu H, He L, Cai J, Huang Q, Cai Q, Li B, Bai Y, Zhang Z, Zhang Y, Wang W, Li J, Wei F, Li H, Jian M, Li J, Zhang Z, Nielsen R, Li D, Gu W, Yang Z, Xuan Z, Ryder OA, Leung FC, Zhou Y, Cao J, Sun X, Fu Y (2010). The sequence and de novo assembly of the giant panda genome. Nature.

[62] Zhang G, Fang X, Guo X, Li L, Luo R, Xu F, Yang P, Zhang L, Wang X, Qi H, Xiong Z, Que H, Xie Y, Holland PW, Paps J, Zhu Y, Wu F, Chen Y, Wang J, Peng C, Meng J, Yang L, Liu J, Wen B, Zhang N, Huang Z, Zhu Q, Feng Y, Mount A, Hedgecock D (2012). The oyster genome reveals stress adaptation and complexity of shell formation. Nature.

[63] Brisson JA, Ishikawa A, Miura T (2010). Wing development genes of the pea aphid and differential gene expression between winged and unwinged morphs. Insect Mol Biol.

[64] Jurka J, Kapitonov VV, Pavlicek A, Klonowski P, Kohany O, Walichiewicz J (2005). Repbase Update, a database of eukaryotic repetitive elements. Cytogenet Genome Res.

[65] **RepeatMasker Open-3.3.0.** [http://www.repeatmasker.org/]

[66] Price AL, Jones NC, Pevzner PA (2005). De novo identification of repeat families in large genomes. Bioinformatics.

[67] Benson G (1999). Tandem repeats finder: a program to analyze DNA sequences. Nucleic Acids Res.

[68] **Sourceforge.** [http://sourceforge.net/projects/glean-gene]

[69] Trapnell C, Pachter L, Salzberg SL (2009). TopHat: discovering splice junctions with RNA-Seq. Bioinformatics.

[70] Trapnell C, Williams BA, Pertea G, Mortazavi A, Kwan G, van Baren MJ, Salzberg SL, Wold BJ, Pachter L (2010). Transcript assembly and quantification by RNA-Seq reveals unannotated transcripts and isoform switching during cell differentiation. Nat Biotechnol.

[71] Bairoch A, Apweiler R (2000). The SWISS-PROT protein sequence database and its supplement TrEMBL in 2000. Nucleic Acids Res.

[72] Zdobnov EM, Apweiler R (2001). InterProScan–an integration platform for the signature-recognition methods in InterPro. Bioinformatics.

[73] Ashburner M, Ball CA, Blake JA, Botstein D, Butler H, Cherry JM, Davis AP, Dolinski K, Dwight SS, Eppig JT, Harris MA, Hill DP, Issel-Tarver L, Kasarskis A, Lewis S, Matese JC, Richardson JE, Ringwald M, Rubin GM, Sherlock G (2000). Gene ontology: tool for the unification of biology. The Gene Ontology Consortium. Nat Genet.

[74] Kanehisa M, Goto S (2000). KEGG: Kyoto Encyclopedia of Genes and Genomes. Nucleic Acids Res.

[75] Lowe TM, Eddy SR (1997). tRNAscan-SE: a program for improved detection of transfer RNA genes in genomic sequence. Nucleic Acids Res.

[76] Griffiths-Jones S, Moxon S, Marshall M, Khanna A, Eddy SR, Bateman A (2005). Rfam: annotating non-coding RNAs in complete genomes. Nucleic Acids Res.

[77] **Rhodnius prolixus.** [http://rhodnius.iq.ufrj.br/]

[78] Li H, Coghlan A, Ruan J, Coin LJ, Hériché JK, Osmotherly L, Li R, Liu T, Zhang Z, Bolund L, Wong GK, Zheng W, Dehal P, Wang J, Durbin R (2006). TreeFam: a curated database of phylogenetic trees of animal gene families. Nucleic Acids Res.

[79] Ruan J, Li H, Chen Z, Coghlan A, Coin LJM, Guo Y, Heriche JK, Hu Y, Kristiansen K, Li R (2008). TreeFam: 2008 update. Nucleic Acids Res.

[80] Guindon S, Gascuel O (2003). A simple, fast, and accurate algorithm to estimate large phylogenies by maximum likelihood. Syst Biol.

[81] Guindon S, Dufayard JF, Lefort V, Anisimova M, Hordijk W, Gascuel O (2010). New algorithms and methods to estimate maximum-likelihood phylogenies: assessing the performance of PhyML 3.0. Syst Biol.

[82] Benton MJ, Donoghue PC (2007). Paleontological evidence to date the tree of life. Mol Biol Evol.

[83] Donoghue PCJ, Benton MJ (2007). Rocks and clocks: calibrating the Tree of Life using fossils and molecules. Trends Ecol Evol.

[84] Rannala B, Yang Z (2007). Inferring speciation times under an episodic molecular clock. Syst Biol.

[85] Yang Z (2007). PAML 4: phylogenetic analysis by maximum likelihood. Mol Biol Evol.

[86] Yang Z, Rannala B (2006). Bayesian estimation of species divergence times under a molecular clock using multiple fossil calibrations with soft bounds. Mol Biol Evol.

[87] **Tracer version 1.4.** [http://beast.bio.ed.ac.uk/software/tracer/]

[88] Xue J, Bao YY, Li B, Cheng YB, Peng ZY, Liu H, Xu HJ, Zhu ZR, Lou YG, Cheng JA, Zhang CX (2010). Transcriptome analysis of the brown planthopper Nilaparvata lugens. PLoS One.

[89] Stamatakis A (2006). RAxML-VI-HPC: maximum likelihood-based phylogenetic analyses with thousands of taxa and mixed models. Bioinformatics.

[90] Jones DT, Taylor WR, Thornton JM (1992). The rapid generation of mutation data matrices from protein sequences. Comput Appl Biosci.

[91] Letunic I, Bork P (2007). Interactive Tree Of Life (iTOL): an online tool for phylogenetic tree display and annotation. Bioinformatics.

[92] Miyamoto T, Slone J, Song X, Amrein H (2012). A fructose receptor functions as a nutrient sensor in the Drosophila brain. Cell.

[93] Dus M, Min S, Keene AC, Lee GY, Suh GS (2011). Taste-independent detection of the caloric content of sugar in Drosophila. Proc Natl Acad Sci U S A.

[94] Fishilevich E, Vosshall LB (2005). Genetic and functional subdivision of the Drosophila antennal lobe. Curr Biol.

[95] Jones WD, Cayirlioglu P, Kadow IG, Vosshall LB (2007). Two chemosensory receptors together mediate carbon dioxide detection in Drosophila. Nature.

[96] Pfaffl MW (2001). A new mathematical model for relative quantification in real-time RT-PCR. Nucleic Acids Res.

[97] Noda H, Omura T (1992). Purification of yeast-like symbiotes of planthoppers. J Invertebr Pathol.

[98] Rose MD, Winston F, Hunter P (1990). Methods in Yeast Genetics: A Laboratory Course Manual.

[99] Quinn NL, Levenkova N, Chow W, Bouffard P, Boroevich KA, Knight JR, Jarvie TP, Lubieniecki KP, Desany BA, Koop BF, Harkins TT, Davidson WS (2008). Assessing the feasibility of GS FLX Pyrosequencing for sequencing the Atlantic salmon genome. BMC Genomics.

[100] Boetzer M, Henkel CV, Jansen HJ, Butler D, Pirovano W (2011). Scaffolding pre-assembled contigs using SSPACE. Bioinformatics.

[101] 101.**Metarhizium acridum.** [http://www.ncbi.nlm.nih.gov/genome/?term=%20Metarhizium%20acridum]

[102] Wang Y, Fan HW, Huang HJ, Xue J, Wu WJ, Bao YY, Xu HJ, Zhu ZR, Cheng JA, Zhang CX (2012). Chitin synthase 1 gene and its two alternative splicing variants from two sap-sucking insects, *Nilaparvata lugens* and *Laodelphax striatellus* (Hemiptera: Delphacidae). Insect Biochem Mol Biol.

[103] **Sequence Read Archive.** [http://www.ncbi.nlm.nih.gov/sra]

