# Supplementary material for: Genomes of the rice pest brown planthopper and its endosymbionts reveal complex complementary contributions for host adaptation
Source: Genome Biol. 2014 Dec 3;15(12):521. doi: 10.1186/s13059-014-0521-0 (PMC4269174; doi:10.1186/s13059-014-0521-0)
Supplement: Additional file 1: — Supplementary Figures S1 to S21 and Tables S1 to S30. [file 13059_2014_521_MOESM1_ESM.pdf]

Additional file for

Genomes of the rice-pest brown planthopper and its endosymbionts reveal complex complementary contributions for host adaptation

Jian Xue[#], Xin Zhou[#], Yan-Yuan Bao[#], Li-Li Yu[#] , Hai-Wei Fan, Zhuo Wang, Hai-Jun Xu, Yu Xi, Zeng-Rong Zhu, Wen-Wu Zhou, Peng-Lu Pan, Bao-Ling Li, John K. Colbourne, Hiroaki Noda, Yoshitaka Suetsugu. Tetsuya Kobayashi, Yuan Zheng, Shanlin Liu, Rui Zhang, Yang Liu, Ya-Dan Luo, Dong-Ming Fang, Yan Chen, Dong-Liang Zhan, Xiao-Dan Lv, Yue Cai, Zhao-Bao Wang, Hai-Jian Huang, Ruo-Lin Cheng, Xue-Chao Zhang, Yi-Han Lou, Bing Yu, Ji-Chong Zhuo, Yu-Xuan Ye, Wen-Qing Zhang, Zhi-Cheng Shen, Huan-Ming Yang, Chuan-Xi Zhang*, Jian Wang*, Jun Wang*, Jia-An Cheng*

[#] These authors contributed equally to this work.

*To whom correspondence should be addressed. Email: chxzhang@zju.edu.cn (C.X.Z.) , wangjian@genomics.cn (J.W.), wangj@genomics.cn (J.W.), jacheng@zju.edu.cn (J.A C.)

This file includes

Table S1 to S30

Figure S1 to S21

Supplementary Tables

Table S1 | Sequencing data for shotgun insert-size libraries.

Pair-end Libraries	Insert Size	Total Data (G)	Read Length (bp)	Sequence Depth (X)	Physical Depth (X)
	180 bp	5.85	100	4.87	4.39
	200 bp	40.16	100	33.47	33.47
	500 bp	75.08	100	62.57	156.42
	2 Kb	2.84	44	2.37	53.79
Illumina Reads	2 Kb	35.49	49	29.58	603.57
	5 Kb	19.60	49	16.33	833.33
	10 Kb	33.56	49	27.97	3424.49
	20 Kb	24.81	49	20.67	4219.39
	40 Kb	26.38	49	21.98	8972.79
Total		263.77		219.81	18301.64

Note: Assuming the genome size is 1.2 Gb.

Table S2 | Clean WGS sequencing data after filtering.

Pair-end Libraries	Insert Size	Total Data (Gbp)	Read Length (bp)	Sequence Depth (X)	Physical Depth (X)
	180 bp	5.12	100	4.27	3.84
	200 bp	35.03	100	29.19	29.19
	500 bp	61.65	100	51.37	128.44
	2 Kb	2.35	44	1.96	44.51
Illumina Reads	2 Kb	24.06	49	20.05	409.18
	5 Kb	9.89	49	8.24	420.49
	10 Kb	8.89	49	7.41	755.95
	20 Kb	4.55	49	3.79	773.81
	40 Kb	6.47	49	5.39	2200.68
Total		158.01		131.67	4766.09

Note: Assuming the genome size is 1.2 Gb.

Table S3 | Statistics for 17-mer analysis.

K-mer	K-mer Num	K-mer depth	Genome Size	Used Bases	Used reads	Depth (X)
17	29,286,217,750	24	1,220,259,072	34,969,752,790	355,220,940	28.66

Table S4 | Sequencing data for Fosmid libraries.

Insert Size	# Libraries	# Lane	Total Data (Gbp)	Clean fosmid sequence data after filtering （Gbp）
250 bp	48,096	23	473.03	262.859
500 bp	48,096	23	452.59	245.081
Total	96,192	46	925.62	507.940

Table S5 | Genome assembly of the BPH.

	Contig		Scaffold	
	Size (bp)	Number	Size (bp)	Number
N90	5,637	44,022	45,095	4,009
N80	9,812	30,820	120,285	2,509
N70	14,155	22,408	201,965	1,777
N60	18,788	16,313	270,831	1,294
N50	24,220	11,642	356,597	928
Maximum length	230,350	----	2,254,159	----
Total length	993817338	----	1,140,786,310	----
Total Number (≥100 bp)	----	80046	----	46,558
Total Number (≥2 kbp)	----	66,371	----	12,347

Table S6 | Gene coverage evaluated with EST data.

Dataset (bp)	Number	Total Length (bp)	Covered by Assembly (%)	With >90% Sequence in one Scaffold		With >50% Sequence in one Scaffold	
				Number	%	Number	%
>0	94,822	28,276,772	95.26	79,507	83.85	90,634	95.58
>200	45,057	21,161,788	96.08	38,919	86.38	43,759	97.12
>500	12,253	11,296,085	95.98	10,253	83.68	11,856	96.76
>1000	3,530	5,339,990	95.59	2,765	78.33	3,381	95.78

Note: The genome coverage was evaluated by EST sequences using BLAT. About 96% of the EST sequences had been covered by our genome assembly.

Table S7 | Statistics of the completeness of *N*. *lugen* and *A*. *pisum* genomes based on 248 core eukaryotic genes (CEG).

	N. lugen		***A. pisum***	
**Type**	**Proteins**	**Completeness (%)**	**Proteins**	**Completeness (%)**
Complete	234	94.35	238	95.97
Group 1	61	92.42	63	95.45
Group 2	53	94.64	52	92.86
Group 3	57	93.44	60	98.36
Group 4	63	96.92	63	96.92
Partial	240	96.77	248	100
Group 1	64	96.97	66	100
Group 2	56	100	56	100
Group 3	57	93.44	61	100
Group 4	63	96.92	65	100

Note: “Complete” refers to CEG proteins covered by our genome assembly with aligned length greater than 70%. “Partial” refers to CEG proteins covered by the assembly with coverage rate exceeded a pre-computed minimum alignment score.

Table S8 | Statistics of the two gene sets overlapped with KOG genes predicted by CEGMA.

Type	BPH gene-set		Pea aphid gene-set	
	Number	Percent (%)	Number	Percent (%)
total KOGs	426	--	455	
one KOGs aligned with one gene	372	87.32	431	94.73
CDS overlap>0.8	180	42.25	412	88.79
CDS overlap>0.5	296	69.48	425	93.41
one KOGs aligned with several genes	12	--	4	
one KOGs aligned with zero gene	42	--	20	

Note: BPH gene-set indicates gene set predicted for *N. lugen*. Pea aphid gene-set indicates gene set downloaded from for *A. pisum*. The statistics result showed how many genes in the gene-set overlapped with predicted KOG (Eukaryotic Orthologous Groups) genes by CEGMA.

Table S9 | General statistics of repeats found in the BPH genome using different programs.

Programs	Repeat Size (bp)	% of genome
TRF	72,580,728	6.36
RepeatMasker	36,382,620	3.19
RepeatProteinMask	89,149,973	7.81
De novo	502,791,303	44.07
Total	554,399,873	48.60

Note：The total repeat percent of genome include simple repeated sequence from the methods of TRF and Denovo.

Table S10 | TE contents in the BPH genome.

	RepBase TEs		TE Proteins		*De novo*		Combined TEs	
	Length (bp)	%in Genome	Length (bp)	% in Genome	Length (bp)	% in Genome	Length (bp)	% in Genome
DNA	16,619,690	1.46	18,316,703	1.61	144,793,819	12.69	162,024,958	14.20
LINE	12,013,992	1.05	56,484,058	4.95	155,726,500	13.65	182,652,892	16.01
SINE	841,429	0.07	0	0.00	7,534,345	0.66	8,272,412	0.73
LTR	8,840,021	0.77	14,352,627	1.26	164,690,704	14.44	168,492,299	14.77
Other	15,304	0.00	0	0.00	26,657	0.00	41,262	0.00
Unknown	0	0.00	0	0.00	21,890,733	1.92	21,890,733	1.92
Total	36,382,620	3.19	89,149,973	7.81	420,285,300	36.84	443,795,677	38.90

Note: Repbase TEs: the result of *RepeatMasker* based on Repbase; TE proteins: the result of *RepeatProteinMask* based on Repbase; *De novo*: Result of *RepeatMasker* by using library predicted through *De novo*; Combined: combined results of Repbase TEs, TE proteins, and *De novo*.

Table S11 | General statistics of predicted protein-coding genes.

Gene set		Number	Average gene length (bp)	Average CDS length (bp)	Average exon per gene	Average exon length (bp)	Average intron length (bp)
De novo	AUGUSTUS	33,881	10231.68	1,129	4.08	277	2956.85
	GENSCAN	80,549	7110.10	811	3.64	223	2385.01
Homolog	*A. pisum*	51,355	2854.20	745	2.09	357	1937.83
	T. castaneum	38,423	4634.33	950	2.53	375	2402.10
	A. mellifera	15,648	8874.66	850	3.90	218	2770.44
	P. humanus	18,663	7695.38	872	3.55	245	2672.62
	H. sapiens	12,750	7142.32	845	3.42	247	2597.95
GLEAN		28,591	10919.04	1,166	4.20	278	3051.66
RNA-Seq		29,090	10995.07	1,152	4.17	276	3048.33
Final		27,571	11400.67	1135.02	4.29	264.43	3061.91

Note: Three approaches were used in gene prediction: Homolog (*A. pisum, T. castaneum, A. mellifera, P. humanus,* and *H. sapiens*), *De novo* (*GENSCAN, AUGUSTUS*), and RNA-seq. The results can be consolidated using the program *GLEAN*.

Table S12 | Summary of evidence for the GLEAN gene models.

	≥20% overlap		≥50% overlap		≥80% overlap	
	**No.**	**Ratio(%)**	**No.**	**Ratio(%)**	**No.**	**Ratio(%)**
P (single)	485	1.76	1,318	4.78	5,413	19.63
P (more)	13,635	49.45	15,467	56.10	14,839	53.82
H (single)	136	0.49	126	0.46	128	0.46
H (more)	234	0.85	212	0.77	199	0.72
P+H	11,729	42.54	8,833	32.04	4,409	15.99

Note: P: *ab initio* prediction; H: homology-based. According to number of gene sources support, the evidence was further separated into single (with one gene source) and more (with two or more gene sources). The overlap threshold is relative to the CDS region of *GLEAN* genes.

Table S13 | Statistics of function annotation.

		**Number**	**Percent (%)**
Total		27,571	--
	InterPro	12,734	46.19
	GO	10,245	37.16
Annotated	KEGG	10,569	38.33
	Swissprot	12,554	45.53
	TrEMBL	15,321	55.57
Unannotated		11,153	40.45

Note: Five protein databases were chosen to assist the function prediction of genes: InterPro, Gene ontology, KEGG, Swissprot, and TrEMBL. The table shows numbers of genes matched in each database.

Table S14 Comparison of predicted *N. lugens* genes with RNA sequence supports.

threshold (alignment length>50)	Unannotated genes (11,153)		Annotated genes (16,418)		Species-specific genes (16,330)	
	Mapped gene number	%	Mapped gene number	%	Mapped gene number	%
Identity>=95	5,549	49.75	14,122	86.02	12,020	73.61
Identity>=98	3,392	30.41	12,531	76.32	9,746	59.68
Identity>=100	1,004	9.00	2,734	16.65	5,278	32.32

Note : All 16,418 annotated, 11,153 unannotated and 16,330 BPH-specific predicted genes were blasted against assembled transcripts (unigene) from the joint eight transcriptome sets, with e-value 1e-5 and length >50bp as threshold.

Table S15 Primers, expected sizes, amplification and sequencing results for the RT-PCR validation of 30 randomly chosen N. lugens-specific genes.

NO.	CDS ID	Primer	Length (bp)	PCR Produ	PCR Produ	Identity%
1	NLU026930.2	sense: 5’-ATGAAGGTTCTTGTTTGC-3’ antisense: 5’-TTAATGGTGGTAGCTGC-3’	912	√	882	99.55
2	NLU027349.1	sense: 5’-ATGGCAGCCTGCAAAAT-3’ antisense: 5’-CTACTTGAAAGCAGCCA-3’	363	√	363	99.17
3	NLU002430.1	sense: 5’-ATGAAAACTTTCTACGC-3’ antisense: 5’-ATCAGTGCAAGTACTGTG-3’	418	√	418	99.28
4	NLU017866.1	sense: 5’-ATGAAGGAGACTGTGCTG-3’ antisense: 5’-TCAACGCTGCTGCTTTC-3’	675	√	675	99.70
5	NLU004347.2	sense: 5’-ATGGCGGTCGGGAAACTAA-3’ antisense: 5’-TTAAGATCTGTTGGCTGGTC-3’	1446	√	1527	95.71
6	NLU027618.1	sense: 5’-ATGTATCACAGCAAAGTG-3’ antisense: 5’-TTAACAAACACGAGCG-3’	636	√	702	96.23
7	NLU023945.1	sense: 5’-ATGAGAGGAATTCAATTAGTTACT-3’ antisense:	624	√	624	98.56
8	NLU017151.1	sense: 5’-ATGAAGGTGAGCAAATG-3’ antisense: 5’-TTAAAATGATAGAAATTCATCACAC-3’	696	√	696	99.71
9	NLU009392.1	sense:5’-ATGGGCTTCTTCACACTGCT-3’ antisense:5’-TCATTGTCTGATCTC-3’	1287	×		
10	NLU010321.1	sense:5’-ATGGTGCATCGGCTAAGA-3’ antisense:5’-TCAGGTCCGAGTAGGTAG-3’	614	√	614	99.84
11	NLU006173.1	sense:5’-ATGAAGACAGCTCTGGTC-3’ antisense:5’-TTACCTTGTCTTCTTCTC-3’	1104	√		
12	NLU018864.1	sense:5’-ATGATTGTAAAAAGATT-3’ antisense:5’-TTAGACGCATCGCAGCG-3’	552	√	552	99.46
13	NLU002431.1	sense:5’-ATGGGTGCAGTCACTCT-3’ antisense:5’-CTAACGTCTGAGTGATCTT-3’	510	√	510	99.41
14	NLU006174.1	sense:5’-ATGGAAGGTTTGAAGGTG-3’ antisense:5’-TCAGATGATGTACGGGCTG-3’	1017	√	1035	99.51
15	NLU007581.1	sense:5’-ATGATTCCAAAAATGCT-3’ antisense:5’-TTAATGGAAGTTACTGTG-3’	798	√	798	99.37
16	NLU007224.1	sense:5’-ATGGGGCTGAGTTGCTT-3’ antisense:5’-TCAGTCTCCTACAACTT-3’	591	√	591	100.0
17	NLU003065.1	sense:5’-ATGTTGTGCTATAGGACAGC-3’ antisense:5’-TCACCAGTAGTGTCCGTAGGG-3’	399	√	387	99.48
18	NLU018884.1	sense:5’-ATGTCTGATATTGCTACTGAAATC-3’ antisense:5’-TTAGACGCCGTTGGTGA-3’	378	√	378	99.47
19	NLU005874.1	sense:5’-ATGCTGGCTACGCGCTTCTCTCT-3’ antisense:5’-TTACCCGTTTCTAGTGGGCCGACA	300	√	300	99.33
20	NLU009669.1	sense:5’-ATGCATTACAGTCTGAT-3’ antisense:5’-TTAGTTCTGTGTGTTCT-3’	693	√		
21	NLU006922.1	sense:5’-ATGGCTATGAGAAGATTTTT-3’ antisense:5’-TCTACTTTGCTTTCTTG-3’	907	√		

22	NLU019613.1	sense:5'-ATGCGCACCAATATGTGGTTC-3'<br>antisense:5'-GAAGGTCAATGACTGGAAGGA-3'	885	√	885	99.66
23	NLU025004.1	sense:5'-ATGAAGGGAATCGTACTGTGTG-3'<br>antisense:5'-TCAACTGAAATCGACGTCTCCA-3'	948	√		
24	NLU021893.1	sense:5'-ATGGCCGCCCTTAAAGCTTTGATC-3'<br>antisense:5'-TTAAGCGTAGACAGCGGGGTGG-3'	444	√	495	93.47
25	NLU021124.1	sense:5'-TCCATTATGGCTCAGATGTCT-3'<br>antisense:5'-GTAACCACTCATGTTTTCTAG-3'	672	√	384	99.74
26	NLU006246.2	sense: 5'-ATGACCAGGACCAATCG-3'<br>antisense: 5'-TCAGTAATAACCATGTAGAC-3'	1539	×		
27	NLU026093.1	sense: 5'-ATGGAGTGGTCGATCAT-3'<br>antisense: 5'-TCAGCTAGGTTTGTCTG-3'	369	×		
28	NLU007225.2	sense:5'-ATGAAGGCTGTCGCCGC-3'<br>antisense:5'-TTAGCAGCAGCATGGGGAGG-3'	813	×		
29	NLU005700.1	sense:5'-ATGGAAGCAGAACCGAA-3'<br>antisense:5'-TTATAGGGACATTTTTGGAT-3'	3069	×		
30	NLU015903.1	sense:5'-ATGCTTGTCGAGAGATCC-3'<br>antisense:5'-TTATTCTTCTCCTTGTCTG-3'	615	×		

* CDSs successfully amplified were indicated with "√", failed amplification with "×".

Table S16 | Non-coding RNA genes in the BPH genome.

Type		Copy	Average length (bp)	Total length (bp)	% of genome
miRNA		372	118.50	44,083	0.0039
tRNA		1,982	73.04	144,759	0.0127
rRNA	*rRNA*	142	199.32	28,304	0.0025
	18S	103	231.29	23,823	0.0021
	28S	12	135.50	1,626	0.0001
	5.8S	0	0.00	0	0.0000
	5S	27	105.74	2,855	0.0003
snRNA	snRNA	*198*	135.54	26,836	0.0024
	CD-box	52	110.38	5,740	0.0005
	HACA-box	0	0.00	0	0.0000
	splicing	146	144.49	21,096	0.0018

Table S17 | Gene orthology comparison among the genomes of 15 arthropod species.

Type	*T. urt*	*D. pul*	*B. mor*	*D. ple*	*A. gam*	*A. aeg*	*D. mel*	*T. cas*	*A. mel*	*C. flo*	*N. vit*	*P. hum*	*R. pro*	*N. lug*	*A. pis*
1:1:1	318	318	318	318	318	318	318	318	318	318	318	318	318	318	318
N:N:N	2,082	1,435	1,801	1,886	1,896	2,367	2,129	2,005	1,634	1,675	1,906	1,579	1,917	2,103	2,018
Diptera	0	0	0	0	423	542	404	0	0	0	0	0	0	0	0
Hemiptera	0	0	0	0	0	0	0	0	0	0	0	0	60	36	36
Hymenoptera	0	0	0	0	0	0	0	0	509	432	459	0	0	0	0
Insect	0	0	103	117	136	192	98	116	96	83	95	83	97	103	102
Lepidoptera	0	0	2,053	2,177	0	0	0	0	0	0	0	0	0	0	0
Others	5,500	8,176	7,183	8,142	8,179	9,818	6,975	8,506	6,918	8,897	8,360	6,495	6,416	8,681	11,207
SD	3,982	12,016	562	1,154	443	763	916	1,385	96	1,653	3,347	113	1,756	7,472	9,221
ND	6,334	8,954	2,596	3,072	1,270	1,986	2,849	4,301	1,089	3,298	2,599	2,181	9,891	8,858	10,365
Total	18,216	30,899	14,616	16,866	12,665	15,986	13,689	16,631	10,660	16,356	17,084	10,769	20,455	27,571	33,267
# Species -specific (SD+ND)	10,316	20,970	3,158	4,226	1,713	2,749	3,765	5,686	1,185	4,951	5,946	2,294	11,647	**16,330**	19,586
# With orthologs	7,900	9,929	11,458	12,640	10,952	13,237	9,924	10,945	9,475	11,405	11,138	8,475	8,808	11,241	13,681
Species -specific%	56.63	67.87	21.61	25.06	13.53	17.20	27.50	34.19	11.12	30.27	34.80	21.30	56.94	**59.23**	58.88
With orthologs%	43.37	32.13	78.39	74.94	86.47	82.80	72.50	65.81	88.88	69.73	65.20	78.70	43.06	40.77	41.12

Table S18 | Expanded and contracted gene family numbers on each branch of the 15 arthropod genomes

Branch	Branch Length	no	Expansions			Contractions			Extinctions				
			Families	Genes	Gene Gain/ Family	Families	Genes	Gene Loss/Fa mily	Families	Genes	Gene Extinct /Famil y	No Change	Avg. Exp.
Da. pulex	363	10,057	3,364	11,504	3.4197	16,236	16,569	1.0205	15,786	15,874	1.0056	6,243	-0.1902
P. humanus	308	7,413	370	480	1.2973	8,145	8,357	1.0260	7,894	7,943	1.0062	6,792	-0.2958
R. prolixus	256	6,711	1,760	3,144	1.7864	8,825	9,151	1.0369	8,596	8,798	1.0235	4,722	-0.2255
N. lugens	256	8,564	2,940	8,251	2.8065	6,947	7,043	1.0138	6,743	6,777	1.0050	5,420	0.0454
N. lugens|R. prolixus	33	15,307	55	101	1.8364	29	30	1.0345	0	0	nan	15,223	0.0027
Ac. pisum	289	9,363	3,670	12,680	3.4550	6,143	6,278	1.0220	5,944	6,017	1.0123	5,494	0.2404
Ac. pisum|N. lugens	19	15,307	32	45	1.4063	10	10	1.0000	0	0	nan	15,265	0.0013
N. lugens|P. humanus	15	15,307	6	7	1.1667	7,790	7,792	1.0003	7,773	7,773	1.0000	15,284	-0.2923
A. mellifera	82	7,952	436	604	1.3853	1,803	2,156	1.1958	1,680	1,974	1.1750	7,393	-0.0583
C. floridanus	82	8,800	916	2,899	3.1649	922	964	1.0456	832	842	1.0120	7,794	0.0727
C. floridanus|A. mellifera	113	9,632	216	357	1.6528	1,442	1,568	1.0874	1,316	1,364	1.0365	9,290	-0.0455
Na. vitripennis	195	8,322	1,444	4,948	3.4266	2,760	2,797	1.0134	2,626	2,645	1.0072	6,744	0.0808
Na. vitripennis|A. mellifera	110	10,948	217	333	1.5346	6,747	6,785	1.0056	6,611	6,618	1.0011	10,595	-0.2423
T. castaneum	292	8,232	1,127	2,990	2.6531	6,545	6,575	1.0046	6,406	6,412	1.0009	6,966	-0.1346
An. gambiae	54	8,177	520	866	1.6654	1,226	1,443	1.1770	1,010	1,043	1.0327	7,441	-0.0217
Ae. aegypti	54	8,582	1,611	2,730	1.6946	659	702	1.0653	605	641	1.0595	6,917	0.0761
Ae. aegypti|An. gambiae	186	9,187	615	1,481	2.4081	4,506	4,522	1.0036	4,399	4,403	1.0009	8,465	-0.1142
D. melanogaster	240	7,331	1,085	2,282	2.1032	6,386	6,455	1.0108	6,255	6,277	1.0035	6,115	-0.1567
D. melanogaster|An. gambiae	28	13,586	68	102	1.5000	9	9	1.0000	0	0	nan	13,509	0.0035

B. mori	120	9,095	584	1,104	1.8904	1,598	1,695	1.0607	1,471	1,541	1.0476	8,384	-0.0222
Da. plexippus	120	9,724	1,124	2,128	1.8932	906	945	1.0431	842	870	1.0333	8,536	0.0444
Da. plexippus|B. mori	149	10,566	521	879	1.6871	3,150	3,188	1.0121	3,020	3,029	1.0030	9,915	-0.0867
B. mori|D. melanogaster	23	13,586	52	70	1.3462	1,060	1,065	1.0047	1,052	1,057	1.0048	13,526	-0.0374
D. melanogaster|T. castaneum	13	14,638	37	50	1.3514	2,921	2,921	1.0000	2,921	2,921	1.0000	14,601	-0.1078
D. melanogaster |A.mellifera	18	17,559	49	66	1.3469	5,527	5,530	1.0005	5,521	5,522	1.0002	17,504	-0.2052
D. melanogaster|N. lugens	40	23,080	37	52	1.4054	2,787	2,812	1.0090	2,763	2,781	1.0065	23,019	-0.1036
N. lugens|Da. pulex	64	25,843	28	37	1.3214	849	957	1.1272	790	838	1.0608	25,756	-0.0345
Te. urticae	427	5,988	1,821	5,024	2.7589	20,854	21,283	1.0206	20,645	20,985	1.0165	3,958	-0.6105

Ac. pisum|N. lugens: the branch lead to the MRCA (most recent common ancestor) node of *Ac. pisum* and *N. lugens* genomes.

Avg. Exp. = (Expansion gene number - Contraction gene number)/Total gene family number

The total gene family number is 26,633.

Table S19 | GO over-representation of gene families expanded on *N. lugens* branch (FDR<0.05, p<=0.00086455)

**GO ID**	**GO description**	**Type**	**Number of genes**	**P-value**
GO:0005622	intracellular	Cellular Component	512	4.58E-181
GO:0008270	zinc ion binding	Molecular Function	323	0
GO:0003677	DNA binding	Molecular Function	103	1.47E-10
GO:0006814	sodium ion transport	Biological Process	98	3.95E-59
GO:0005272	sodium channel activity	Molecular Function	98	2.71E-66
GO:0000786	nucleosome	Cellular Component	90	5.39E-63
GO:0006334	nucleosome assembly	Biological Process	90	3.42E-61
GO:0004386	helicase activity	Molecular Function	55	0
GO:0008234	cysteine-type peptidase activity	Molecular Function	35	1.39E-15
GO:0003779	actin binding	Molecular Function	26	2.01E-09
GO:0048037	cofactor binding	Molecular Function	25	2.55E-14
GO:0003964	RNA-directed DNA polymerase activity	Molecular Function	25	4.18E-12
GO:0016043	cellular component organization	Biological Process	25	6.68E-17
GO:0030036	actin cytoskeleton organization	Biological Process	25	1.67E-15
GO:0000036	acyl carrier activity	Molecular Function	25	1.33E-18
GO:0006278	RNA-dependent DNA replication	Biological Process	25	4.18E-12
GO:0031177	phosphopantetheine binding	Molecular Function	22	4.97E-20
GO:0006352	transcription initiation, DNA-dependent	Biological Process	16	3.12E-10
GO:0016986	transcription initiation factor activity	Molecular Function	16	1.29E-13
GO:0015074	DNA integration	Biological Process	11	3.29E-07
GO:0016705	oxidoreductase activity, acting on paired donors, with incorporation or reduction of molecular oxygen	Molecular Function	8	0.0001815

GO:0016702	oxidoreductase activity, acting on single donors with incorporation of molecular oxygen, incorporation of two atoms of oxygen	Molecular Function	8	3.35E-08
GO:0004656	procollagen-proline 4-dioxygenase activity	Molecular Function	8	3.35E-08
GO:0031418	L-ascorbic acid binding	Molecular Function	8	0.0001815

Note: we calculated p-values by Fisher exact test for each GO category. We also corrected P-values by false discovery rate (FDR) considering the multiple testing on all the GO terms.

Table S20 | Gene family contraction (gene loss) analysis results

Family ID (Treefam)	Family function	Number of genes in each species in the family														
		A. pis	***N. lug***	***R. pro***	***P. hum***	***N. vit***	***C. flo***	***A. mel***	***D. mel***	***A. aeg***	***A. gam***	***B. mor***	***D. ple***	***T. cas***	***D. pul***	***T. urt***
**Gene families significantly contracted in *N. lugens* branch**																
1147	ubiquitin carboxyl-terminal hydrolase 10	38	0	39	0	0	0	0	0	0	0	0	0	2	0	0
6029	Probable cytochrome P450	6	2	8	7	18	8	3	22	25	25	14	15	23	6	29
6030	Probable cytochrome P450	24	11	33	10	45	39	31	34	91	40	27	29	68	10	10
**Gene families significantly contracted both in *N. lugens* and *A. pisum* branches**																
1182	trypsin	0	1	2	7	31	4	7	53	50	49	20	20	15	1	0
6029	Probable cytochrome P450	6	2	8	7	18	8	3	22	25	25	14	15	23	6	29
6030	Probable cytochrome P450	24	11	33	10	45	39	31	34	91	40	27	29	68	10	10
**Gene families contracted in branch lead to MRCA (most recent common ancestor) of *Nlug, Rpro* and *Apis***																
1180	tripartite motif-containing protein	0	0	0	4	51	8	3	24	35	22	8	11	7	0	0
1182	trypsin	0	1	2	7	31	4	7	53	50	49	20	20	15	1	0
1188	chymotrypsin	0	1	1	1	1	1	1	29	33	37	21	21	48	18	0
1632	carboxylesterase	18	6	8	5	30	20	16	21	42	29	53	44	36	11	0
3001	Pupal cuticle protein	5	7	10	7	13	13	12	23	42	25	27	19	27	40	0
3838	4-coumarate--CoA ligase	2	1	0	5	9	4	2	13	23	11	8	6	14	4	0
5849	carbonic anhydrase	0	0	0	2	2	2	1	2	2	2	2	2	2	1	4
6029	Probable cytochrome P450	6	2	8	7	18	8	3	22	25	25	14	15	23	6	29
6927	carboxypeptidase A, invertebrate	0	0	0	1	8	2	2	12	18	9	14	13	5	4	0
10096	triacylglycerol lipase	7	6	3	4	23	15	4	23	31	13	21	21	18	16	45

A. pis.,, *Acyrthosiphon pisum*; ***N.lug***, *Nilaparvata lugens*; ***R. pro.,*** *Rhodnius prolixus;* ***P. hum.,*** *Pediculus humanus;* ***N. vit.*** *Nasonia vitripennis*.***, C. flo.,*** *Camponotus floridanus;* ***A. mel.,*** *Apis mellifera*; ***D. mel,*** *Drosophila melanogaster*; ***A. aeg.,*** *Aedes aegypti*; ***A. gam.,*** *Anopheles gambiae*; ***B. mor***., *Bombyx mori*; ***D. ple.,*** *Danaus plexippus;* ***T. cas***., *Tribolium castaneum*; ***D. pul*** ., *Daphnia pulex;* ***T. urt.,*** *Tetranychus urticae.*

Table S21 | The common lost genes in *N. lugens* and *A. pisum*

KEGG annotation (detailed)	Swissprot annotation (detailed)	Gene number
--	Trypsin-1	6
--	Trypsin-1	1
trypsin	Trypsin-2	1
trypsin	Trypsin-3	2
--	Trypsin-3	1
trypsin	Trypsin 3A1	2
trypsin	Trypsin-4	1
trypsin	Trypsin 5G1	1
trypsin	Trypsin-7	4
trypsin	Trypsin alpha	1
trypsin	Trypsin alpha-4	1
trypsin	Trypsin beta	1
trypsin	Trypsin delta/gamma	4
trypsin	Trypsin epsilon	4
trypsin	Trypsin zeta	2
trypsin	Trypsin iota	3
trypsin	Trypsin theta	1
--	Trypsin eta	3
trypsin	Trypsin	4
--	Trypsin I-P1	2
trypsin	Vitellin-degrading protease	3
trypsin	Mite allergen Der p 3	1
trypsin	Hypodermin-B	5
trypsin	Granzyme M (Fragment)	1
kallikrein 7	Trypsin-1	1
kallikrein 14	Kallikrein-14	1
--	Serine protease SP24D	6
--	Serine protease 33	1
--	Serine protease ami	1
chymotrypsin	Serine proteases 1/2	19
chymotrypsin	Serine protease 3	3
chymotrypsin	Chymotrypsin BI	3
chymotrypsin	Chymotrypsinogen 2	1
chymotrypsin	Collagenase	3
--	Chymotrypsin	1
tripartite motif-containing protein 2/3	Chymotrypsin-1	4
tripartite motif-containing protein 2/3	Chymotrypsin-2	7
4-coumarate--CoA ligase	Luciferin 4-monooxygenase	9
4-coumarate--CoA ligase	Probable 4-coumarate--CoA ligase 3	4
esterase / lipase	Lipase 1	3
triacylglycerol lipase	Lipase 1	7

triacylglycerol lipase	Lipase 3	13
carboxypeptidase A, invertebrate	Zinc carboxypeptidase A 1	6
carboxypeptidase A, invertebrate	Zinc carboxypeptidase A 1	4
carboxypeptidase A, invertebrate	Carboxypeptidase B	1
carboxypeptidase B	Carboxypeptidase B	1
**cytochrome P450, family 4, subfamily**		
Cyp4e1	Probable cytochrome P450 4e1	1
Cyp4ad1	Probable cytochrome P450 4ad1	1
Cyp4d21	Probable cytochrome P450 4d21	1
Cyp4ac1	Probable cytochrome P450 4ac1	1
Cyp4d8	Cytochrome P450 4d8	1
Cyp4ac2	Probable cytochrome P450 4ac2	1
Cyp4p1	Cytochrome P450 4p1	1
Cyp4d1	Cytochrome P450 4d1	1
Cyp4d14	Probable cytochrome P450 4d14	1
Cyp4ae1	Cytochrome P450 4ae1	1
Cyp4c3	Cytochrome P450 4c3	1
Cyp4ac3	Probable cytochrome P450 4ac3	1
Cyp4p2	Probable cytochrome P450 4p2	1
Cyp4p3	Probable cytochrome P450 4p3	1
Cyp4d20	Probable cytochrome P450 4d20	1
Cyp316a1	Probable cytochrome P450 316a1	1
Cyp311a1	Probable cytochrome P450 311a1	1
Cyp4e2	Cytochrome P450 4e2	1
Cyp4d2	Cytochrome P450 4d2	1
Cyp4s3	Probable cytochrome P450 4s3	1
Cyp312a1	Probable cytochrome P450 312a1	1
Cyp4e3	Cytochrome P450 4e3	1

The detoxification- and digestion-related genes of two hemiptera insect species were compared with the orthologous genes of the *D. melanogaster*.

Table S22 | Comparison of midgut peritrophic martrix related genes in insect genomes

	Diptera		Lepidoptera	Coleoptera	Hymenoptera	Hemiptera	
	D.m	*A.g*	*B.m*	*T.c*	*A.m*	*A.p*	*N.l*
Peritrophin 1	6	9	2	4	1	0	0
Peritrophin 44	5	3	0	1	0	0	0
Peritrophin 48	8	1	1	0	1	0	0
Peritrophin 55	2	0	0	0	0	0	0
**Peritrophin Total**	**21**	**13**	**3**	**5**	**2**	**0**	**0**
**Chitin Synthase 2**	1	1	1	1	1	0	0

Table S23 | Genome features of the yeast-like symbiont (YLS) and bacterial symbiont, *Arsenophonus nilaparvatae*.

**Features**	**YLS**	***A. nilaparvatae***
Total Length (Mbp)	26.81	2.96
Total scaffold Num (#)	582	20
GC content (%)	55.29	37.59
N50	310,669	199,718
Max Length (bp)	873,274	379,331
Gene number	7156	2,762
Gene Length (bp)	14,598,071	2,455,986
Gene Length / Genome (%)	54.44	83.11
Gene Average Length (bp)	2040	888

Table S24| YLS genes contributing to essential amino acid synthesis.

Amino acid biosynthetic pathway	geneID	ko_name	ko_EC
**Arginine**	A6808	E2.3.1.1	2.3.1.1
	A0158	Arg5	2.7.2.8/1.2.1.38
	A3321	ArgD	2.6.1.11
	A7130	ArgE	3.5.1.16
	A4625	ArgF	2.1.3.3
	A6650,A6652	ArgG	6.3.4.5
	A1074	ArgH	4.3.2.1
**Isoleucine+Valine**	A2441,A6705	IlvH	2.2.1.6
	A6504	IlvC	1.1.1.86
	A6923,A3697	IlvD	4.2.1.9
	A4061,A1798,A0703	IlvE	2.6.1.42
	A6482	IlvA	4.3.1.19
**leucine**	A2441,A6705	IlvH	2.2.1.6
	A6504	IlvC	1.1.1.86
	A6923,A3697	IlvD	4.2.1.9
	A7156	LeuA	2.3.3.13
	A5663	Leu1	4.2.1.33
	A1891	LeuB	1.1.1.85
	A4061,A1798,A0703	IlvE	2.6.1.42
	A1463,A6804	LeuS	6.1.1.4
**lysine**	A4858	E2.3.3.14	2.3.3.14
	A5257	E4.2.1.36	4.2.1.36
	A2828	E1.1.1.87	1.1.1.87
	A5184	Aro8	2.6.1.57
	A2817,A4622,A4641,A6169	E1.2.1.31L	1.2.1.31
	A0073	E1.5.1.10	1.5.1.10
	A0851	E1.5.1.7	1.5.1.7
**Methionine**	A2530	LysC	2.7.2.4
	A1713	Asd	1.2.1.11
	A4109	E1.1.1.3	1.1.1.3
	A3629,A5678	MetX	2.3.1.31
	A5081,A0723,A0724	MetB	2.5.1.48
	A7143	MetC	4.4.1.8
	A3318	MetE	2.1.1.14
**Phenylalanine**	A2312,A2313,A3660,A4600	AroF	2.5.1.54
	A0931	AroB	4.2.3.4
	A5406	AroD	4.2.1.10
	A4377	ARO1	1.1.1.25/2.7.1.71/2.5.1.19

	A4399	AroC	4.2.3.5
	A6839	PheA	5.4.99.5
	A5184	Aro8	2.6.1.57
**Threonine**	A2530	LysC	2.7.2.4
	A1713	Asd	1.2.1.11
	A4109	E1.1.1.3	1.1.1.3
	A0933	ThrB	2.7.1.39
	A2924	ThrC	4.2.3.1
**Tryptophan**	A2312,A2313,A3660,A4600	AroF	2.5.1.54
	A0931	AroB	4.2.3.4
	A5406	AroD	4.2.1.10
	A4377	ARO1	1.1.1.25/2.7.1.71/2.5.1.19
	A4399	AroC	4.2.3.5
	A2457	TrpE	4.1.3.27
	A4949	TrpD	2.4.2.18
	A4989	Trp1	4.1.1.48/5.3.1.24
	A3684	TrpB	4.2.1.20
**Histidine**	A3117	hisA	5.3.1.16
	A0375	hisH	2.4.2.-
	A3873	hisB	4.2.1.19
	A1454,A2860	hisC	2.6.1.9
	A5066	E3.1.3.15	3.1.3.15
	A4772	hisD	1.1.1.23

Table S25 | Genes involved in nitrogen recycling and ammonia assimilation pathways

Species	geneID	ko_EC	Protein description
YLS	A5602	1.7.3.3	uricase
	A0324	3.5.2.17	putative transthyretin-like protein
	A6182,A5317	3.5.2.5	allantoinase
	A5965	3.5.3.4	allantoicase
	A2413	3.5.1.5	urease
	A4253	3.5.3.19	ureidoglycolate hydrolase
	A2520,A2521	1.4.1.4	glutamate dehydrogenase (NADP+)
	A1621	1.4.1.13	glutamate synthase (NADPH/NADH)
	A0308,A3818,A5768,A5826	6.3.1.2	glutamine synthetase
BPH	NLU006642.1	1.7.3.3	uricase
	NLU020864.1	4.1.1.-	2-oxo-4-hydroxy-4-carboxy-5-ureidoimidazoline decarboxylase
	NLU000910.1	1.4.1.4	glutamate dehydrogenase
	NLU000946.1	6.3.1.2	glutamine synthetase

Table S26 | Genes involved in steroid biosynthesis pathway

Species	geneID	ko_EC	Protein description
YLS	A1835	2.5.1.21	farnesyl-diphosphate farnesyltransferase
	A2908	1.14.99.7	squalene monooxygenase
	A1272	5.4.99.7	lanosterol synthase
	A7153	1.14.13.70	cytochrome P450, family 51 (sterol 14-demethylase)
	A4191	1.3.1.70	delta14-sterol reductase
	A3620	1.14.13.72	methylsterol monooxygenase
	A3908	1.1.1.170	sterol-4alpha-carboxylate 3-dehydrogenase
	A0752	1.1.1.270	3-keto steroid reductase
	A1363	2.1.1.41(ERG6)	sterol 24-C-methyltransferase
	A2563	5.-.-.-(ERG2)	C-8 sterol isomerase
	A4727	1.3.3.-(ERG3)	C-5 sterol desaturase
	A4628	1.14.14.-(ERG5)	C-22 sterol desaturase
	A0344	1.3.1.71(ERG4)	delta24(24(1))-sterol reductase
BPH	NLU002609.1, NLU012376.1, NLU019231.1, NLU009526.1, NLU008304.1, NLU022827.1	1.3.1.21	7-dehydrocholesterol reductase, putative
	NLU001141.1	2.1.1.41	sterol 24-C-methyltransferase
	NLU025181.1	1.3.1.72	similar to 24-dehydrocholesterol reductase
	NLU006651.2	5.3.3.5	cholestenol delta-isomerase
	NLU023532.1	1.14.21.6	lathosterol oxidase
	NLU023532.1	1.3.1.21	lathosterol oxidase

Table S27 | *Arsenophonus nilaparvatae* genes contributing to B-vitamins synthesis.

B-vitamins biosynthetic pathway	geneID	ko_EC
Riboflavin	ArsenophonusGL000213	3.5.4.25
	ArsenophonusGL000318	3.5.4.26
	ArsenophonusGL000318	1.1.1.193
	ArsenophonusGL001222	3.1.3.-
	ArsenophonusGL001042	4.1.99.12
	ArsenophonusGL000846, ArsenophonusGL000319, ArsenophonusGL000331, ArsenophonusGL002080, ArsenophonusGL002582	2.5.1.-
	ArsenophonusGL001973	2.5.1.9
Nicotinic acid	ArsenophonusGL000670	2.7.7.18
	ArsenophonusGL001188, ArsenophonusGL001622	3.1.3.5
	ArsenophonusGL000261	2.4.2.1
	ArsenophonusGL000174	3.5.1.19
Pantothenate	ArsenophonusGL000944	3.5.2.2
	ArsenophonusGL000579	3.5.1.6
	ArsenophonusGL001213	6.3.2.1
Pyridoxine	ArsenophonusGL002325	1.2.1.72
	ArsenophonusGL001211	1.1.1.290
	ArsenophonusGL002443	2.6.1.52
	ArsenophonusGL000278	4.2.3.1
Biotin	ArsenophonusGL001488	2.3.1.47
	ArsenophonusGL001486	2.6.1.62
	ArsenophonusGL000240, ArsenophonusGL001490	6.3.3.3
	ArsenophonusGL001487	2.8.1.6
Lipoic acid	ArsenophonusGL000661	2.8.1.8
	ArsenophonusGL000662	2.3.1.181
Folate	ArsenophonusGL002015, ArsenophonusGL002152	2.6.1.85
	ArsenophonusGL001109	4.1.3.38
	ArsenophonusGL000855	2.5.1.15
	ArsenophonusGL001206	6.3.2.12/6.3.2.17
	ArsenophonusGL001054	1.5.1.3

Table S28 | Wing development network genes.

Function	Gene names	BPH ID	*Drosophila* ID	pea aphid
a/p	*en*	NLU020892.1	AAF58639.1	ACYPI002874
	hh	NLU028412.1	AB 66186.	ACYPI006525
		NLU028416.1		
	ci	NLU020108.	AF59373.2	ACYPI45156
	ptc	NLU003813.1	AAF59062.1	ACYPI000356
	dpp	NLU027538.1	AAN10434.1	ACYPI004615
				ACYPI009598
				ACYPI009127
				ACYPI25043
	Dad	NLU027365.3	AAX52959.1	ACYPI009993
	brk	NLU018299.1	AAF46251.3	ACYPI47548
	salm	NLU003510.1	AAF53097.3	ACYPI003922
D/V	*ap*	NLU018087.2	AAM68357.2	ACYPI000502
		NLU009649.1		ACYPI006883
		NLU024135.1		
		NLU009648.1		
	N	NLU022806.1	AAF45848.2	ACYPI005150
		NLU022804.1		
	Ser	NLU027786.1	AAF56678.2	ACYPI003637
		NLU019675.1		
	wg	NLU010606.1	AAN10628.1	ACYPI003251
		NLU010333.1		
	Dll	NLU020890.1	AAF47280.1	ACYPI36103
	vg	NLU013430.1	AAF58444.1	ACYPI34460
Bodywall/wing	*hth*	NLU005530.1	AAX52943.2	ACYPI008722
	tsh	NLU025765.1	AAS64735.1	ACYPI000453
	nub	NLU025618.1	AAF53205.2	ACYPI005847
		NLU006167.1		
	vvl	NLU025618.1	AAF50641.3	ACYPI008866
		NLU015807.1		
Hox	*Ubx*	NLU001543.1	AAS65158.1	ACYPI001856
others	*spi*	NLU023548.2	AAZ66477.1	ACYPI33281
	sna	NLU027752.1	AAF53463.1	N/A
	sd	NLU020346.1	AAS65351.1	ACYPI001652
		NLU014285.1		
	sc	NLU023528.1	AAF45499.1	ACYPI000805
	exd	NLU014004.1	AAN09379.1	ACYPI009075
		NLU010828.1		
	esg	N/A	AAF53458.1	ACYPI001198
	ct	NLU017429.1	AAF46264.2	ACYPI003171

bi	NLU001602.1	AAF45946.2	ACYPI000143
Antp	NLU006941.1	AAS65110.1	ACYPI008076
ac	NLU023528.1	AAF45498.1	N/A
abd	NLU016183.1	AAF55360.2	ACYPI009147

Table S29 | Primers designed for real-time PCR of selected chemoreception genes.

Gene	Primers	Sequence (5'-3')
NlCSP1	F	CGAAAGCTGCTGCCAAAATT
	R	GTTCCTCCATCAGGTGCTTGA
NlCSP6	F	GCCAAAGTGATGGCGTTCAT
	R	ACATGTACTTTGCGCGGAAAA
NlCSP7	F	TCAAATGCCTCACCGATAAGG
	R	GGACTGCATTTGGTGCATGA
NlCSP8	F	GATGTCAATTCGCTGCTCAA
	R	TTCGATCGGGATCATACACA
NlCSP10	F	CCTGATGCAATCCAGTCCAA
	R	AGTCGCGCTCCCTACTTTGA
NlCSP11	F	ATAAGCCCTGCGACCATGTT
	R	TGATCTTCCTCCCGTTGTGAA
NlCSP12	F	TCCTCCTCGTCTGCCTTCTG
	R	TCGTCGAGGTCGATGTTGTC
NlCSP13	F	AGAAGGATGCACTCCTGAAGGA
	R	CGCTGCCGTTTTCTGTTTG
NlCSP14	F	TGCCAGATGCCCTTGCA
	R	TCTTTGGGTCGCTTCTCGAT
NlCSP16	F	GTGGCGGTTTTGTGCTTTTC
	R	GCTGGTTGCTGAGCACTTCA
NlOBP4	F	GCTGAACGTGGAAGCTTTGAA
	R	TTCCATGCATTTGTCAACGATT
NlOBP8	F	GGACTTCAAGCCAGACAATGA
	R	TGGGGCATTTTTCAAGAGTC
NlOBP9	F	TGGCTCAAATTTTCCGTCTTTC
	R	GGATTCAACTCCTACGACATGCT
NlOBP11	F	AACCCAAATGCCCATGATTC
	R	CAGCAGTGGAAGGTAGTCGTTAAG
NlOR43(83b)	F	GGGTGATGTCTGTGTCTGGA
	R	ATAGGCCAACAGGGTGAGTG
NlGR1	F	AGAGGAGAGAAGCCAAAGCC
	R	TGGCCTTCGACACATCATTA
NlGR2	F	TCGAATCCGTCAAAGGAAAG
	R	AGGAGAACAATGAGCGAGAGA
NlGR5	F	TGGTCAACCGAATTACAACAA
	R	TTCCTGTATCGTAGGCTGGC
NlGR7	F	AGCCTCCAACAATGGACAAC
	R	GCGTGAAATTTAGTGCGCTT
alpha-tubulin	F	ACGTCCTTGGGAACGACATC
	R	GCTTTGAGCCAGACAACCAAA

Table S30 | Primers designed for wing development network genes

Genes	Primers	sense
Nlabd-A	F	TTGGGCCTATCATTACCA
	R	CACTTCATTCGCCTGTTC
Nlspi	F	AAGACAGCGTGTAGCGAGTA
	R	GGTCACCTCCACCATCCA
Nlbi	F	CCGCCTACCAGAATGAG
	R	TCGAGTCGTCCTGAAGC
Nlen	F	CTCACCTCCAGAAATGC
	R	CTTCAGGCGAGACAGC
Nltsh	F	TGGTAGGAGGAGACAAA
	R	TTCCAGCAGAATGGAGT
Nlwg	F	ATAACCTGCTACCCTTGTCA
	R	GTTCCACCTCCTGTTTCTG
Nldll	F	CGTACCACCCTTACCAGC
	R	TTGCCTTTGCCATTGTT
Nlexd	F	TCCAGGTTCCTCGATGCC
	R	CGATACTTGTGAAACGGTGAT
Nlptc	F	AAACTGCTCCCAACAAAC
	R	GACACCTCCGAGAATAAGA
Nlvg	F	AGCAACTACCAGAGCACCAA
	R	AGCAACAGGCTGCCATAC
Nlhth	F	CCACCAACATACTGCG
	R	TGGTTGAACGATACGC
Nlsc	F	GCAAGCGGAGAATCAGTTT
	R	GTCACCGACACGGGGATG
Nlsd	F	ACCAACATACAGGACGAGAC
	R	CTGGCAGATGCTTGAGCT
Nlvvl	F	GCTCCTGTCGTCTCCTCA
	R	CGGGTGTTGGTGGTTGT
NlN	F	ACGATAAGGACGAAACCC
	R	TCCAATAGCCGCACAAT
Nlci	E	ACGGGAGGTGGTGGATT
	R	CTGACATTGGAGTCGCTGA
Nlcut	F	CACCGAATGCCAACCAA
	R	CCTGCGATAGTCCAAGAA
Nlap	F	TGGAGGTGGCGTTTGGC
	R	GCGTCAGGGTTATGGTTG
Nldpp	F	TCGCCGACCATCTCAAC
	R	CACAGCCTTGCACAGTCAT
Nlhh	F	TTGCCGCTTCGGGTTTG
	R	GCGTGTTCAGGCGTTCT
Nlsalm	F	AATGTATGTGGCAGTCGG
	R	GAGCAATCAGAGGAGGGT

Supplementary figures

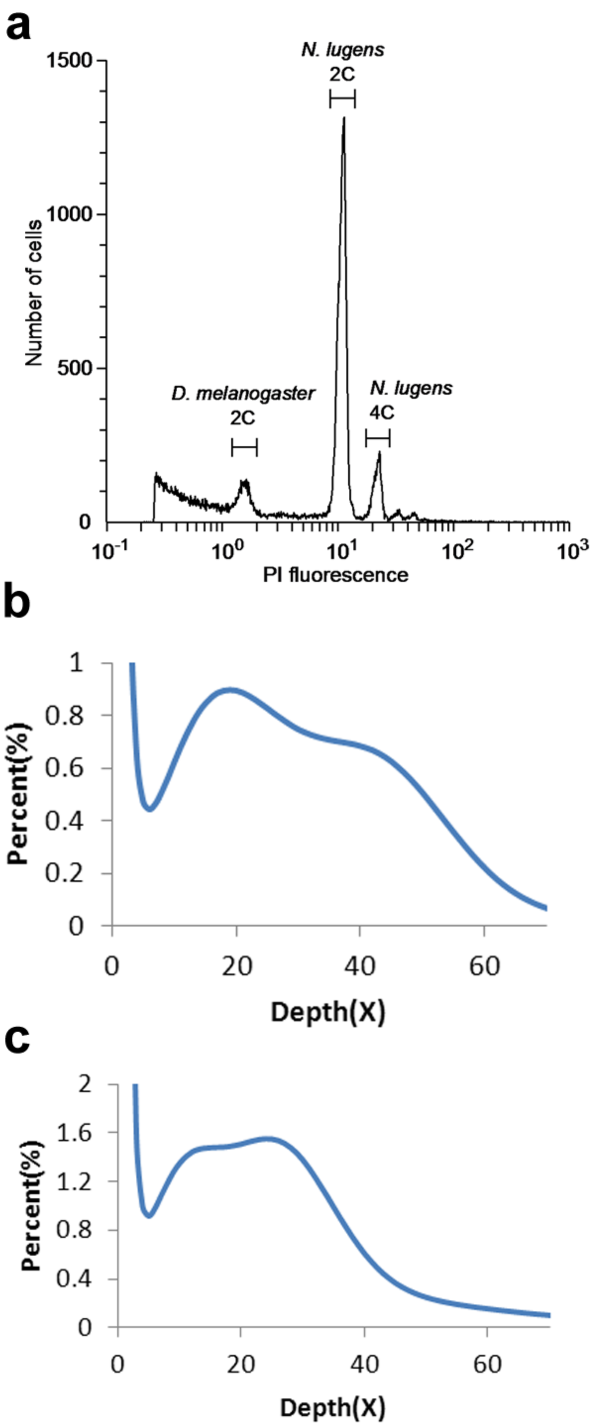

Figure S1 Estimations for genome size and heterozygosity of the BPH genome

a. Genome size estimation using flow cytometry. Histogram of fluorescent signals from nuclei stained with propidium iodide (PI). The sample shows *D. melanogaster* 2C peak and *N. lugens* adult female 2C and 4C peaks. The C value (haploid nuclear DNA content) of *N. lugens* adult female was estimated to be 1.14 pg. Genome sizes of female and male was estimated to be 1,110 Mbp and 1,137 Mbp, respectively. The same procedure revealed that the genome sizes of *Bombyx*

mori and *Locusta migratoria* were 463 Mbp and 6.7 Gbp, respectively. b-c. Genome size and heterozygosity estimations using the k-mer approach. 17-mer (b.) and 27-mer (c.) estimations of the BPH genome size. The X-axis represents the sequencing depth (X), and the Y-axis represents the proportion of K-mers of a given sequencing depth of the total K-mer numbers. For genomes with low heterozygosis, the 17-mer distribution will obey a Poisson distribution. When the heterozygosis level is not trivial, a sub-peak will occur at half of the depth of the main peak (a), and the sub-peak will rise when performing 27-mer analysis (b) compared to 17-mer analysis.

.

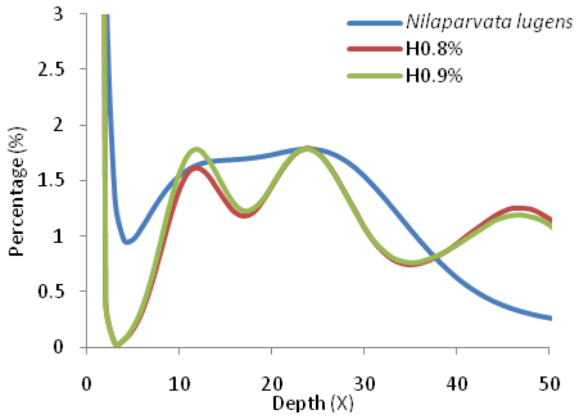

Figure S2 Calculating the heterozygosis level of the BPH genome using the 17-mer method.

In the 17-mer analysis, we found that the heterozygous level was high (~0.8%). The real 17-mer distribution curve is generally close to the H0.8% simulation curve (17-mer distribution generated with the heterozygous rate set at 0.8% in a simulation analysis).

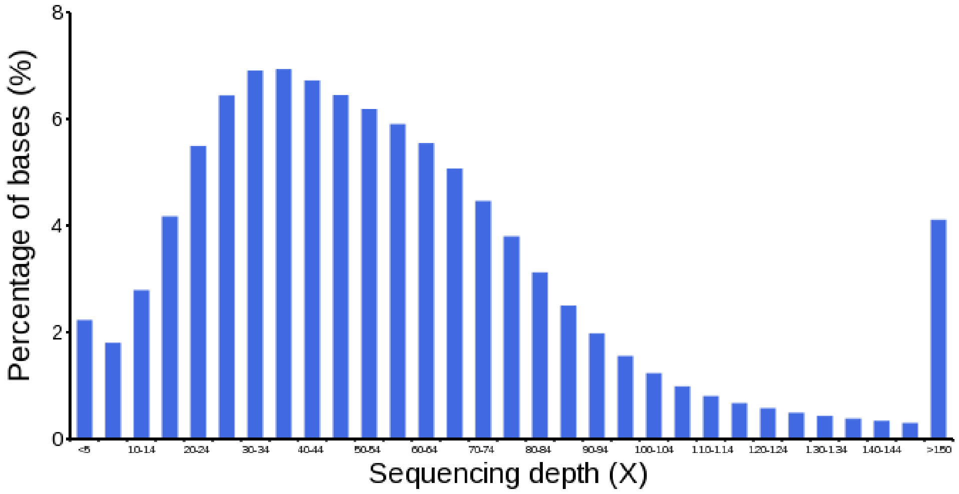

Figure S3 Sequence depth distribution.

The X-axis represents sequencing depths, and the Y-axis represents the proportion of base numbers at a given sequencing depth.

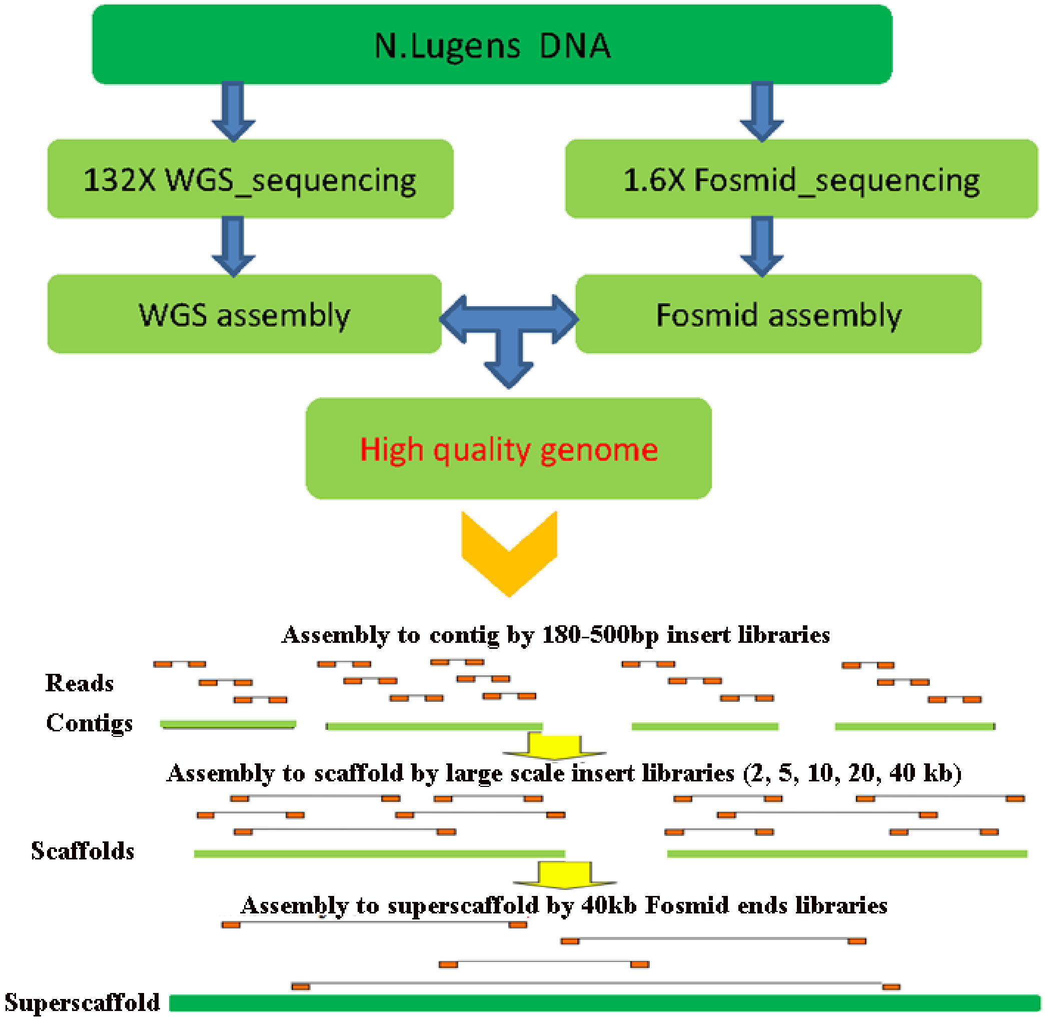

Figure S4 Schematic illustration of the hybrid assembly pipeline combining assemblies from WGS and fosmids.

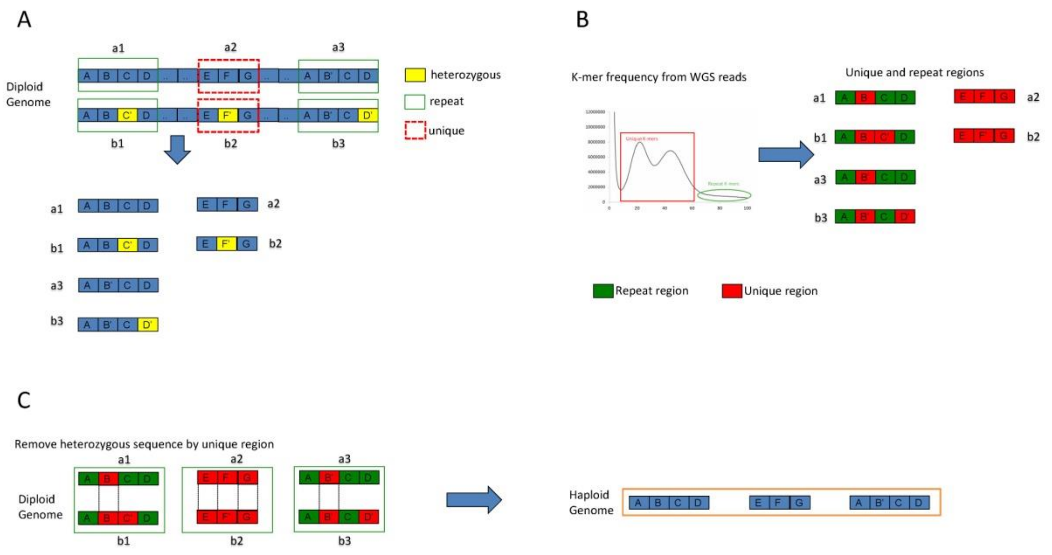

Figure S5 The process of remove heterozygous sequences

A.A diploid genome contains repeat regions(it may be include allele genes) and unique regions(that contain sequences that only occur once in a haploid genome), as show in the top region.The Rabbit software only combine the sequences with similarity >95% and leave all sequences with low similarity in library, as show below. B.We build a math model to distinguish heterozygous sequences and dupplication.Using the WGS reads to account the occurrence of k-mer and distinguish repeat and unique regions. C. For a haploid genome, a unique region should occur only once in the genome. If two sequences have the same unique region, we remove the shorter one and keep the longer.

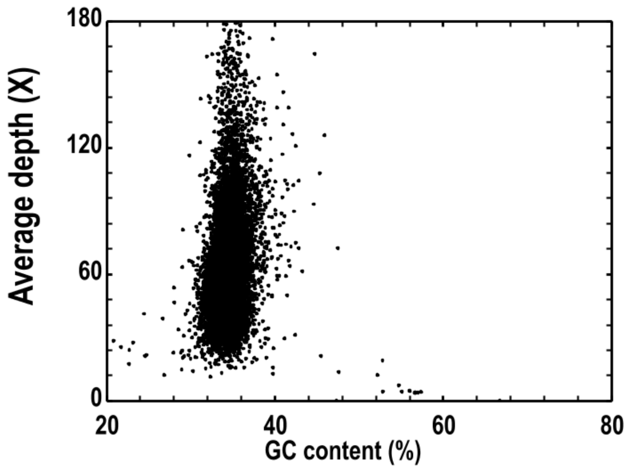

Figure S6 GC content and sequencing depth.

We used sliding windows of 500 bp bins (with a 250 bp overlap in between) to calculate GC contents along the genome. The X-axis represents GC contents in percentages, and the Y-axis represents the average depth of a given GC percentage. Each point represents the GC content and corresponding sequencing depth of a particular sliding window.

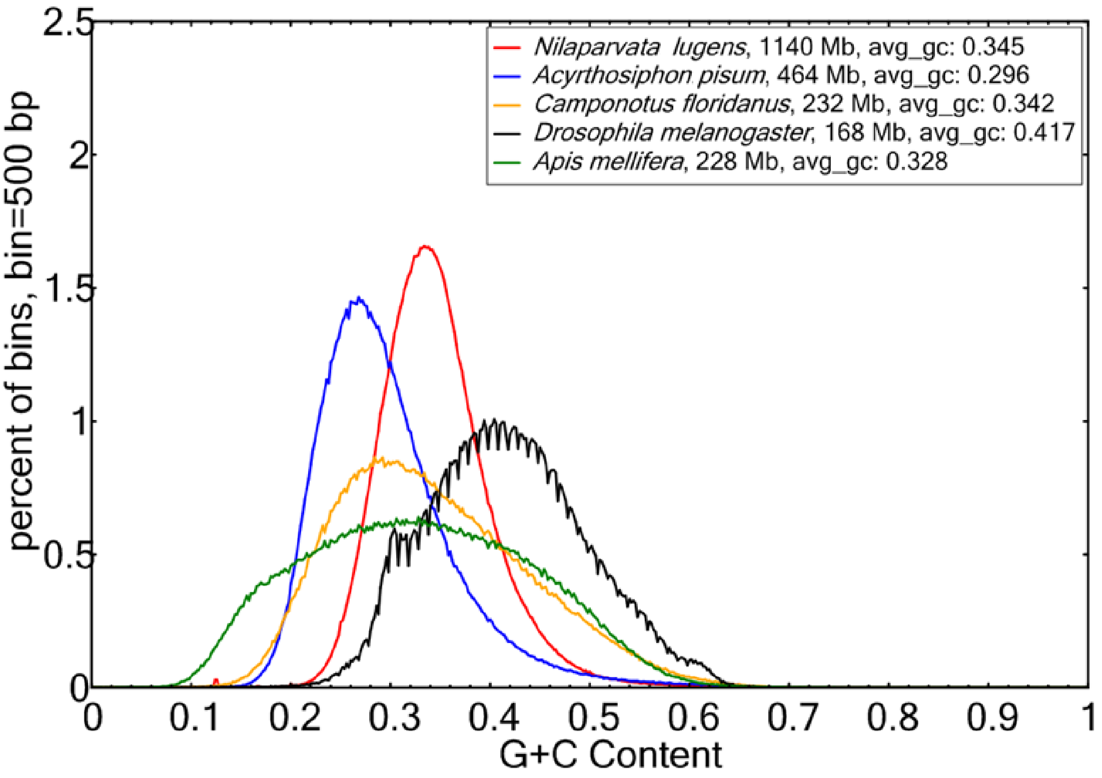

Figure S7 GC content distributions for 5 insect species.

The X-axis represents GC contents, and the Y-axis represents the proportion of bin numbers divided by the total windows. We compared the GC content distribution of *N. lugens* to those in 4 other insect species. *N. lugens* shared a similar GC content distribution pattern with *A. pisum*, with a more condensed distribution on both sides of the average GC contents (34.6% and 29.6% for *N. lugens* and *A. pisum*, respectively). The 2 hymenopteran insects shared similar GC content distributions, with more even distribution on both sides of their average GC contents (34.2% and 32.8% for *C. floridanus* and *A. mellifera*, respectively). The only dipteran species *D. melanogaster* showed a different GC content distribution, with a higher average GC content (41.7%) and intermediate evenness.

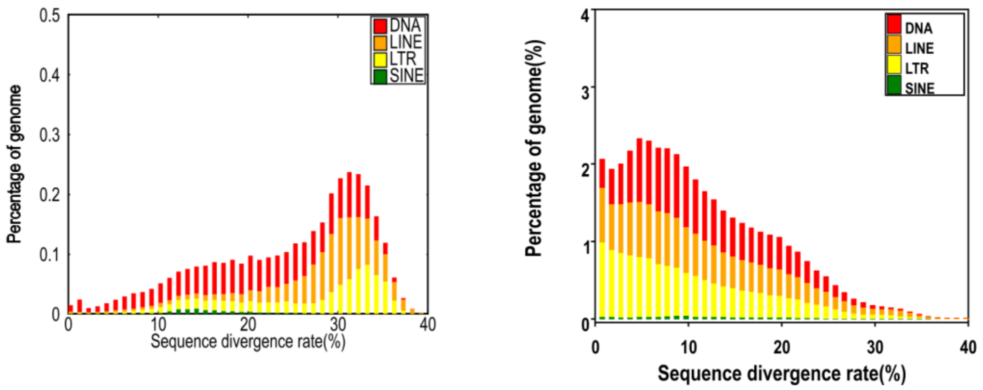

a b

Figure S8 Distribution of the divergence rates for each TE type predicted by both homology-based and *de novo* approaches.

a. TEs were identified from the BPH genome by performing searches for homologous repeats against Repbase using *RepeatMasker*. b. Repetitive sequences were first *de novo* identified using *repeatmodeler* and then compared against Repbase. Transposable elements (TEs) represent one of several types of mobile genetic elements. TEs are assigned to one of two classes according to their mechanism of transposition, which can be described as either copy and paste (class 1 TEs) or cut and paste (class 2 TEs).Class 1 inludes LINE(long- interspersed nuclear elements),SINE(hort interspersed nuclear elements),and LTR(Long terminal repeat retrotransposons).Class 2 includes DNA transposons.

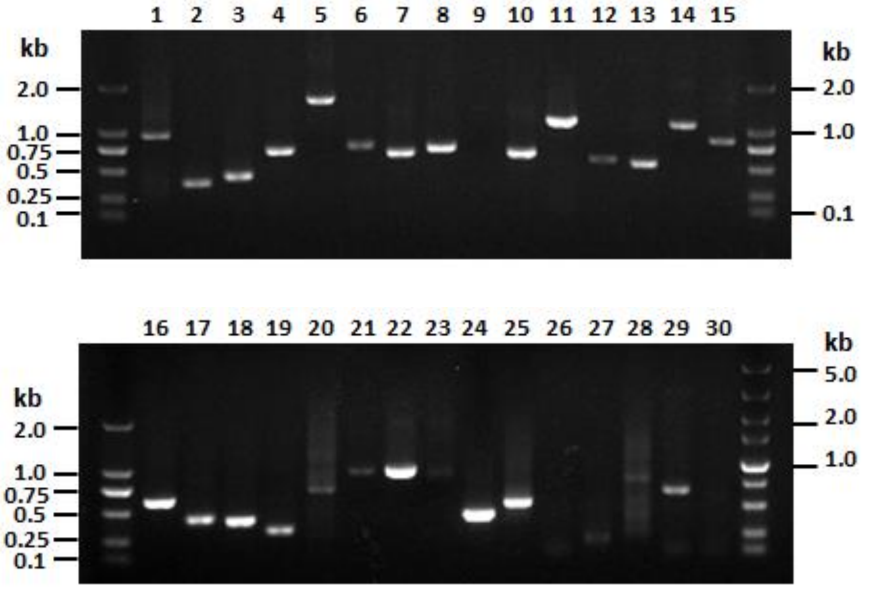

Fig. S9 Validation of 30 randomly chosen *N. lugens*-specific genes by RT-PCR. Twenty-four CDSs with expected sizes were successfully amplified (1-8, 10-25), see Table ? with "√", six CDSs failed to be amplified (Lanes 9, 26, 27, 28, 29, 30). Gene list see Table S10.

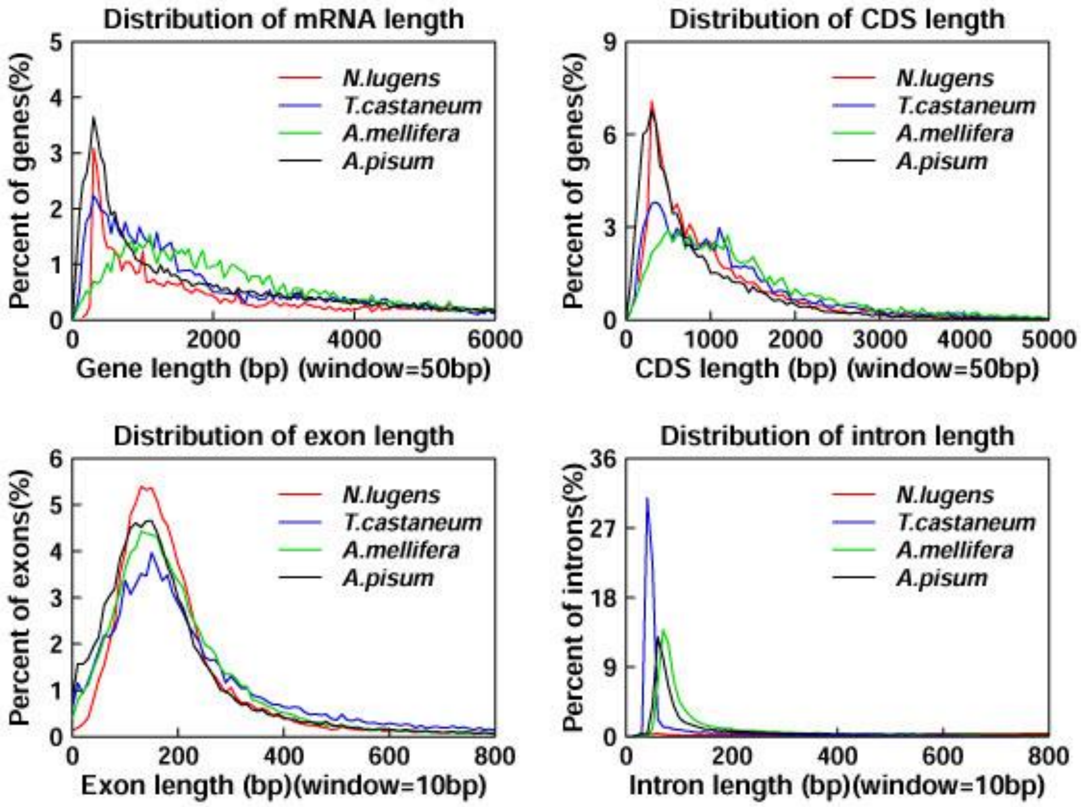

Figure S10 Distributions of gene lengths for the whole gene set, coding DNA sequence (CDS), exons, and introns of 4 insect species.

The percentage was calculated for each sequence length category represented by the window number given in each figure plate.

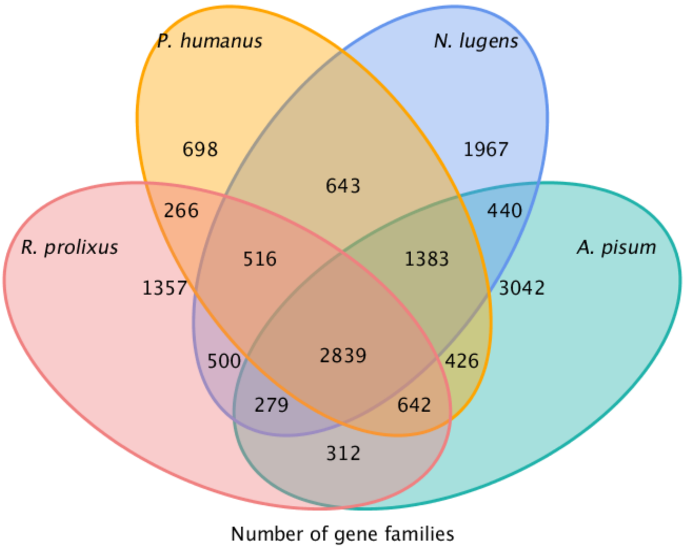

Figure S11 Venn diagram showing the orthologous groups shared among the genomes of 4 insect species, *N. lugens*, *R. prolixus*, *A. pisum*, and *P. humanus*.

There are 2,882 conserved gene families shared among the 4 insect genomes. *N. lugens* has the second most abundant species-specific gene families, following *A. pisum*. The number in parentheses is the gene counts.

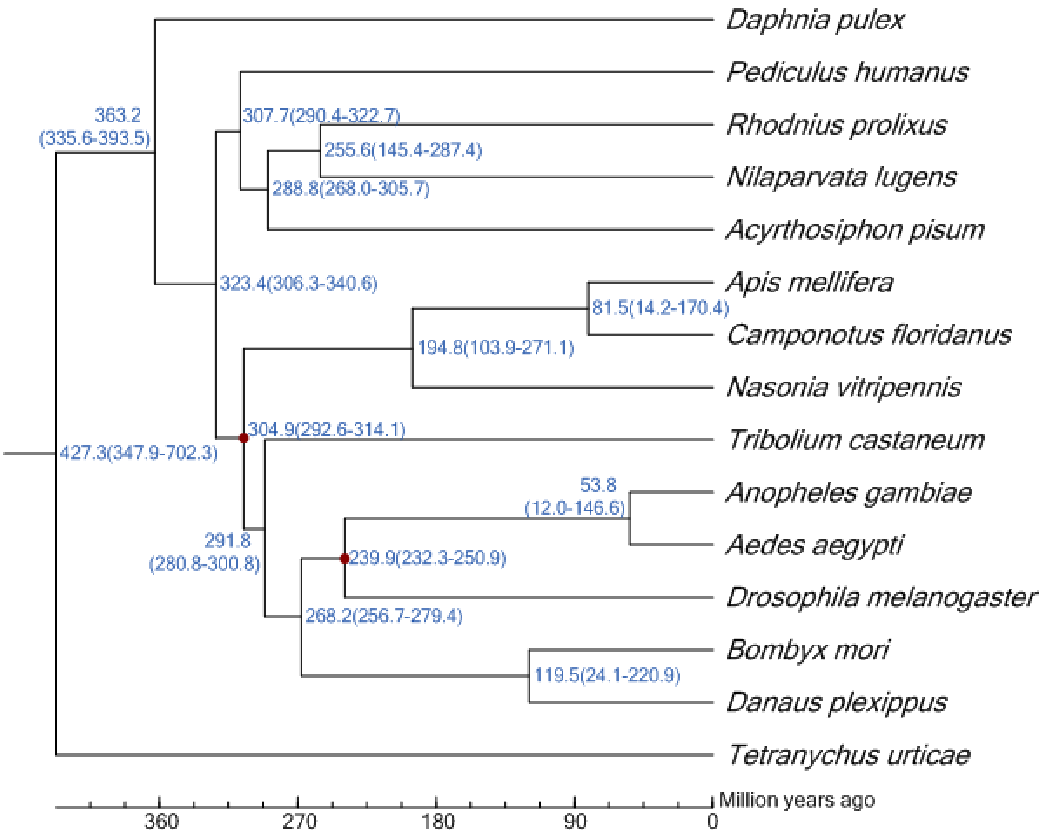

Figure S12 Estimated divergence times among insect genomes using PAML *mcmctree*

Note: The number on each node represents the divergence time from the present (million years ago, Mya), with 95% CI values noted in brackets. The following calibration times were used in the estimation: *A. gambiae-D. melanogaster* divergence (238.5~295.4 Mya), *N. vitripennis-D. melanogaster* divergence (238.5~307.2 Mya).

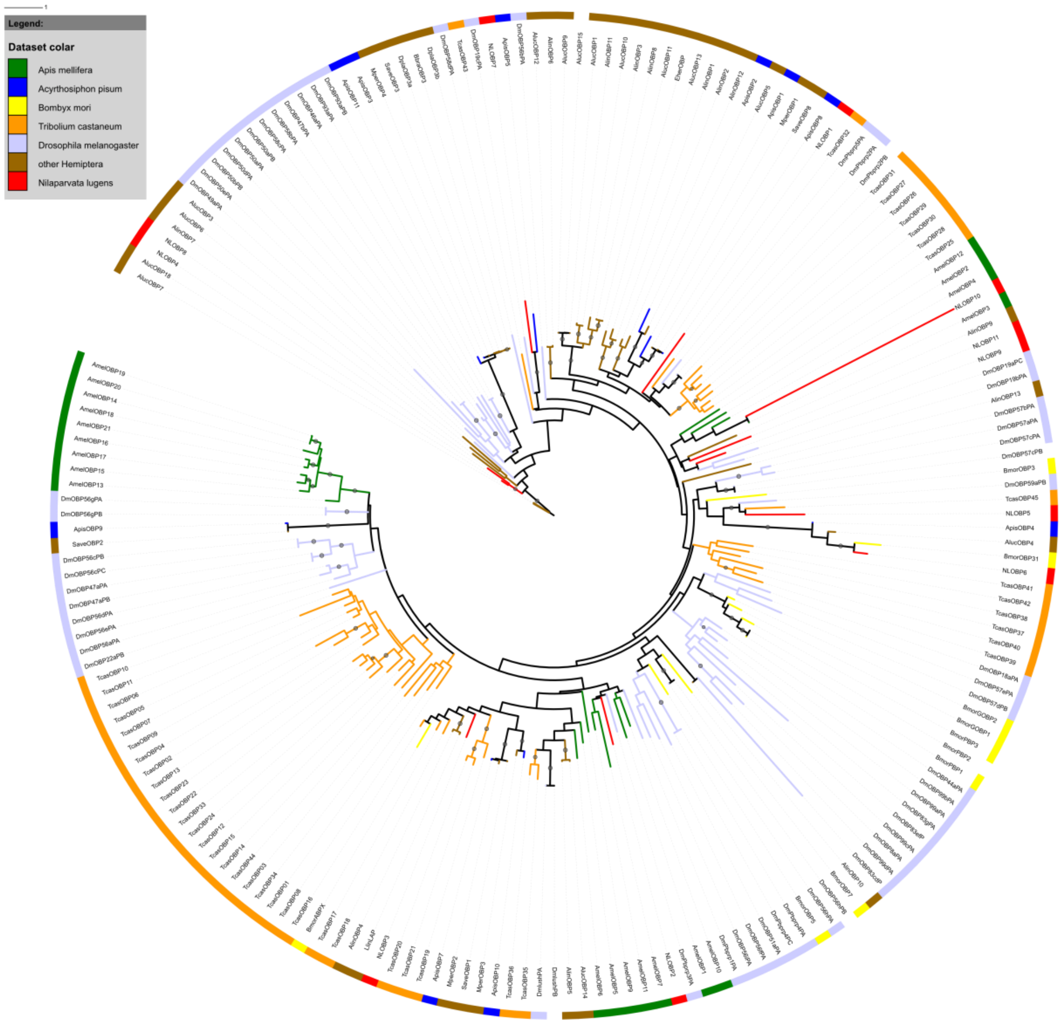

Figure S13 Phylogenetic relationships of OBP proteins from different insect species.

Apis mellifera (Amel), *Bombyx mori* (Bmor), *Drosophila melanogaster* (Dm), *Acyrthosiphon pisum* (Apis), *Tribolium castaneum* (Tcas), *Brevicoryne brassicae* (Bb), *Adelphocoris lineolatus* (Alin), *Euschistus heros* (Eher), *Sitobion avenae* (Save), *Apolygus lucorum* (Aluc), *Myzus persicae* (Mper), *Drepanosiphum platanoidis* (Dpla), *Nilaparvata lugens* (Nl). Genes from the same species are indicated by the same colour. All hemipteran species, except for *N. lugens*, are marked in brown.

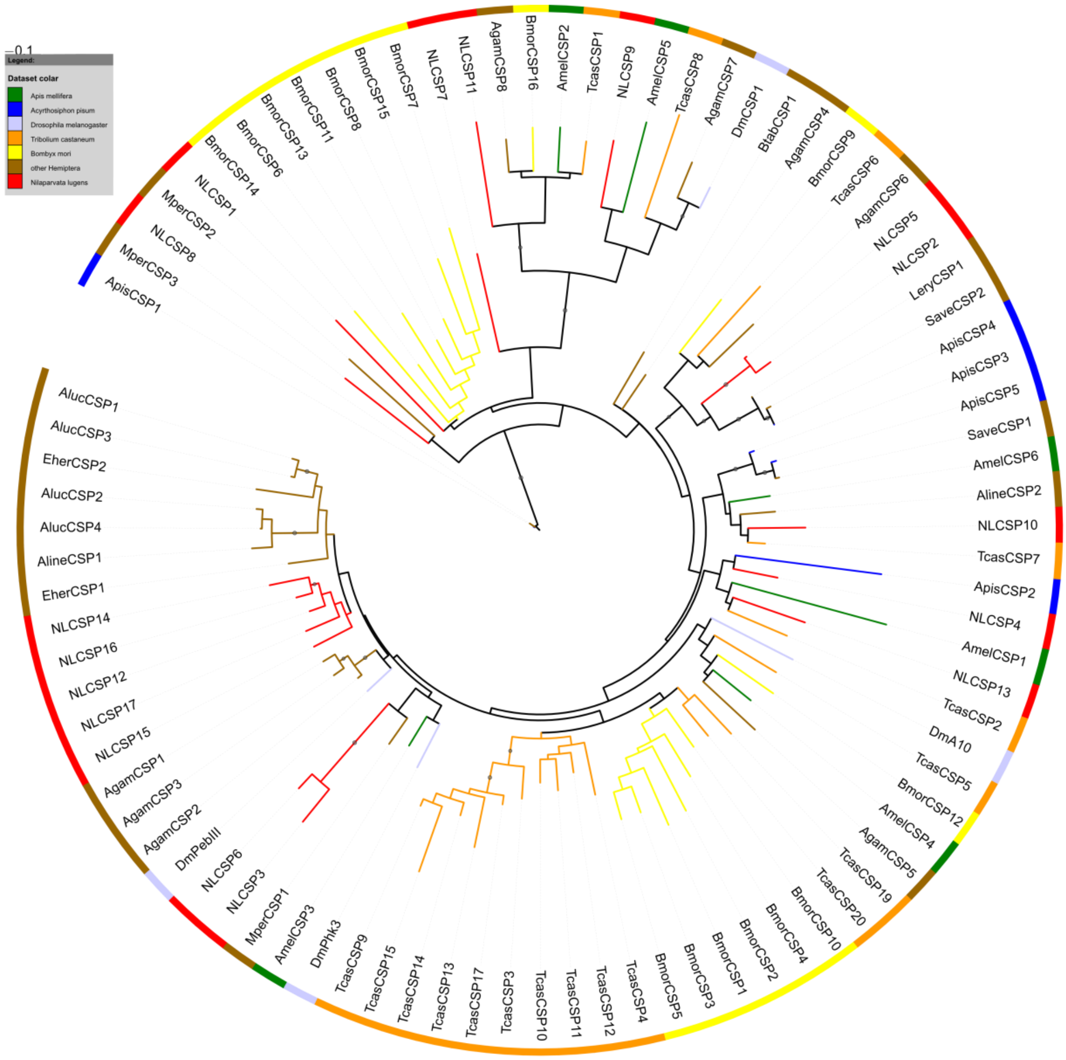

Figure S14 Phylogenetic relationships of CSP proteins from different insect species.

Apis mellifera (Amel), *Bombyx mori* (Bmor), *Drosophila melanogaster* (Dm), *Acyrthosiphon pisum* (Apis), *Tribolium castaneum* (Tcas), *Anopheles gambiae* (Agam), *Adelphocoris lineolatus* (Aline), *Euschistus heros* (Eher), *Sitobion avenae* (Save), *Myzus persicae* (Mper), *Lipaphis erysimi* (Lery), *Nilaparvata lugens* (Nl). Genes from the same species are indicated by the same colour. All hemipteran species, except for *N. lugens*, are marked in brown.

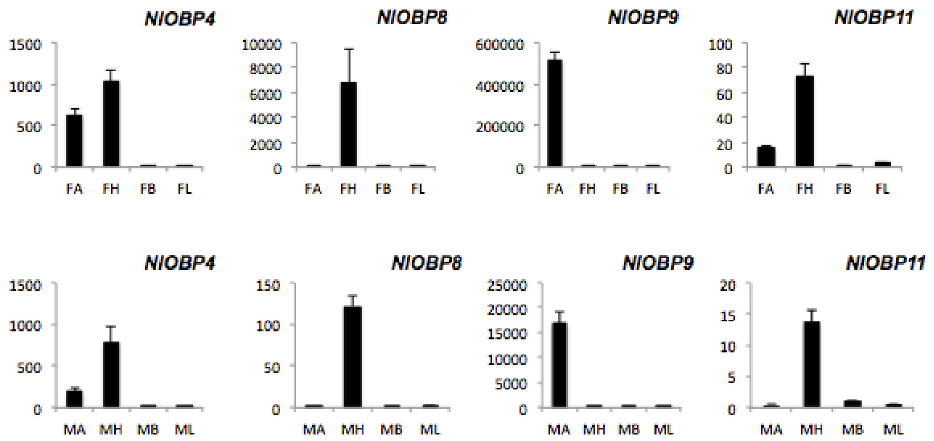

Figure S15 Constitutive transcription profiles of 4 *N. lugens* OBPs across different tissues.

The following tissues were analysed: female adult antennae (FA), female adult head (FH), female adult remaining body (i.e., without head and legs) (FB), female adult legs (FL), male adult antennae (MA), male adult head (MH), male adult remaining body (i.e., without head and legs) (MB), and male adult legs (ML). Transcription levels are expressed as the mean fold difference in transcription relative to the female adult remaining body or male adult remaining body.

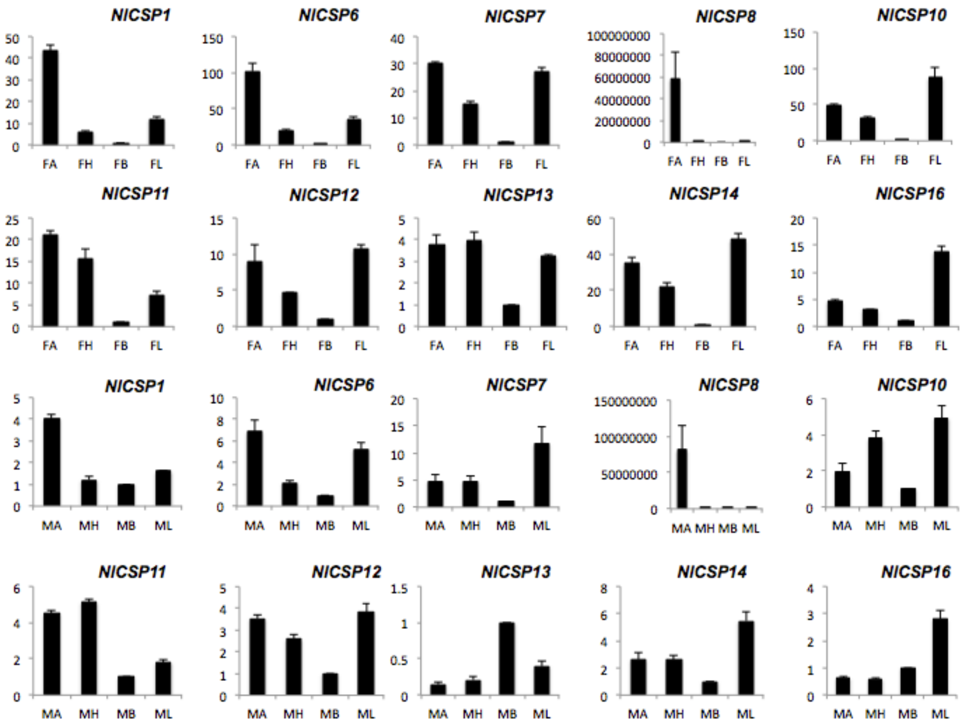

Figure S16 Constitutive transcription profiles of 10 *N. lugens* CSPs across different tissues.

The following tissues were analysed: female adult antennae (FA), female adult head (FH), female adult remaining body (i.e., without head and legs) (FB), female adult legs (FL), male adult antennae (MA), male adult head (MH), male adult remaining body (i.e., without head and legs) (MB), and male adult

legs (ML). Transcription levels are expressed as the mean fold difference in transcription relative to the female adult remaining body or male adult remaining body.

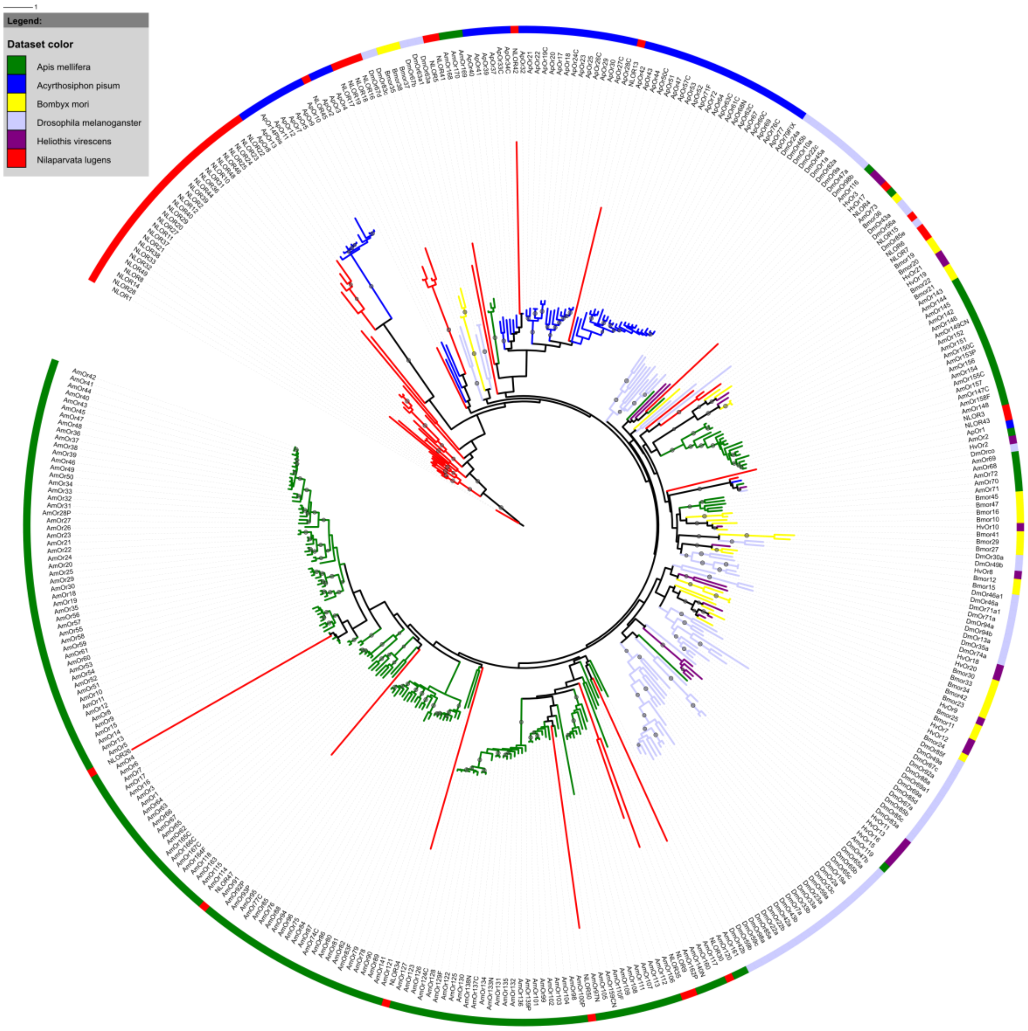

Figure S17 Phylogenetic relationships of OR proteins from different insect species.

Drosophila melanogaster (Dm), *Bombyx mori* (Bm), *Heliothis virescens* (Hv), *Acyrthosiphon pisum* (Ap), *Apis mellifera* (Am), *Nilaparvata lugens* (NLU). Genes from the same species are indicated by the same colour.

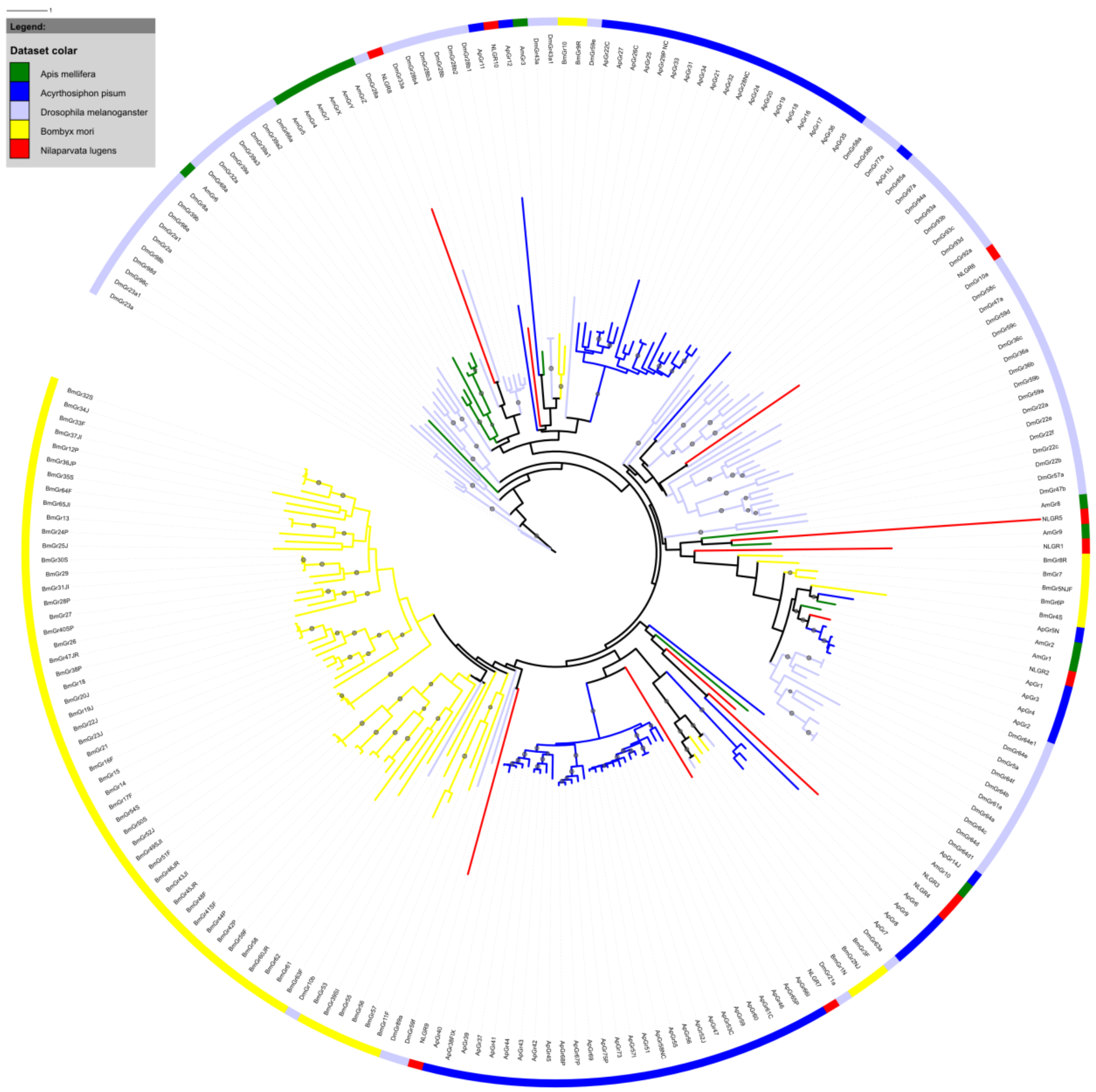

Figure S18. Phylogenetic relationships of GR proteins from different insect species.

Drosophila melanogaster (Dm), *Acyrthosiphon pisum* (Ap), *Nilaparvata lugens* (NLU), *Apis mellifera* (Am) and *Bombyx mori* (Bm). Genes from the same species are indicated by the same colour.

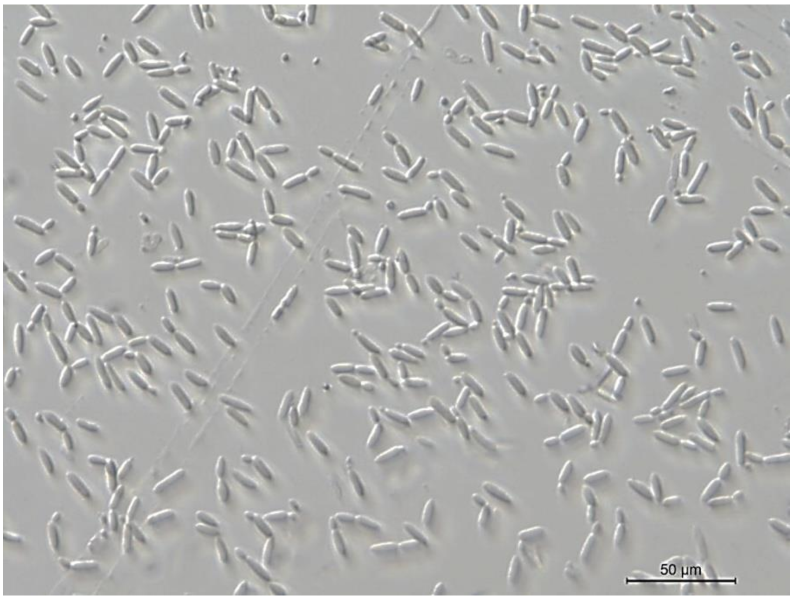

Figure S19 Microscopic images of the yeast-like symbionts isolated from *N. lugens*.

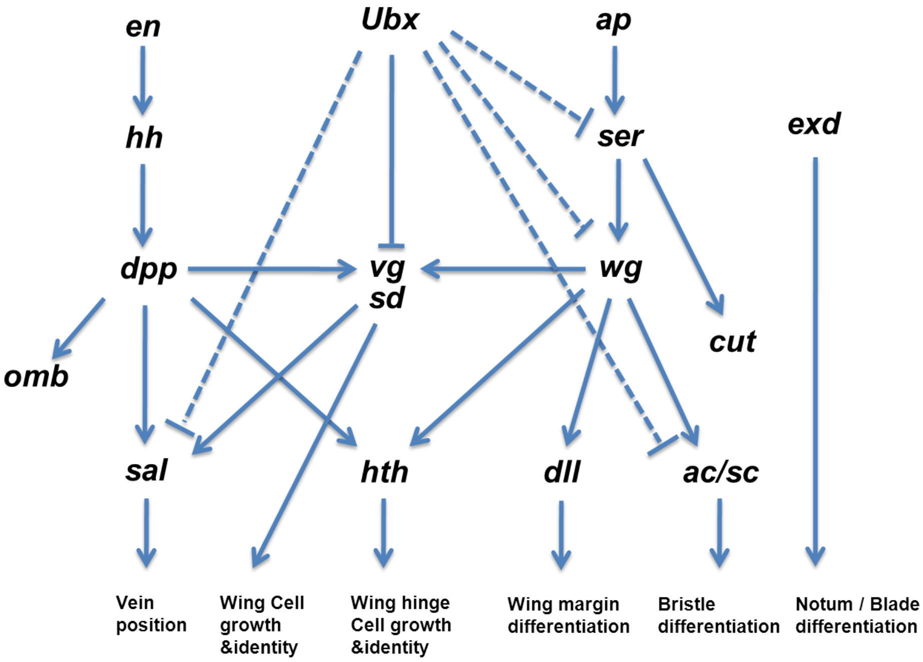

Figure S20. Wing development network deduced from relevant genes from *Drosophila*

Schematic diagram of the wing developmental network in BPH. Solid line with arrow represents activation; solid line with bar presents repression; dashed line with bar represents regulations specific to pea aphid

a

Gene names	2nd	3rd	4th	5th
abd	0.60	1.56	0.79	0.64
spi	3.25	5.53	4.56	2.17
bi	3.65	3.77	2.51	1.72
en	2.55	3.63	5.19	2.60
tsh	6.07	8.90	7.33	13.31
wg	8.04	13.05	12.92	7.82
du	1.65	2.47	2.09	1.37
exd	1.20	2.57	2.20	1.79
ptc	1.24	1.09	2.28	2.00
vg	1.64	2.47	3.61	2.63
hth	2.02	3.62	2.53	1.86
sc	6.20	10.68	10.72	7.55
sd	1.67	3.17	2.93	2.25
vvl	5.35	5.54	5.51	2.07
N	1.80	2.88	2.52	1.87
Ci	39.37	31.97	19.79	11.61
Cut	1.46	2.52	2.23	1.90
ap	26.90	57.49	54.42	14.70
dpp	4.02	5.89	4.80	3.60
hh	1.46	2.84	2.26	1.74
salm	3.33	6.00	4.61	4.07

b

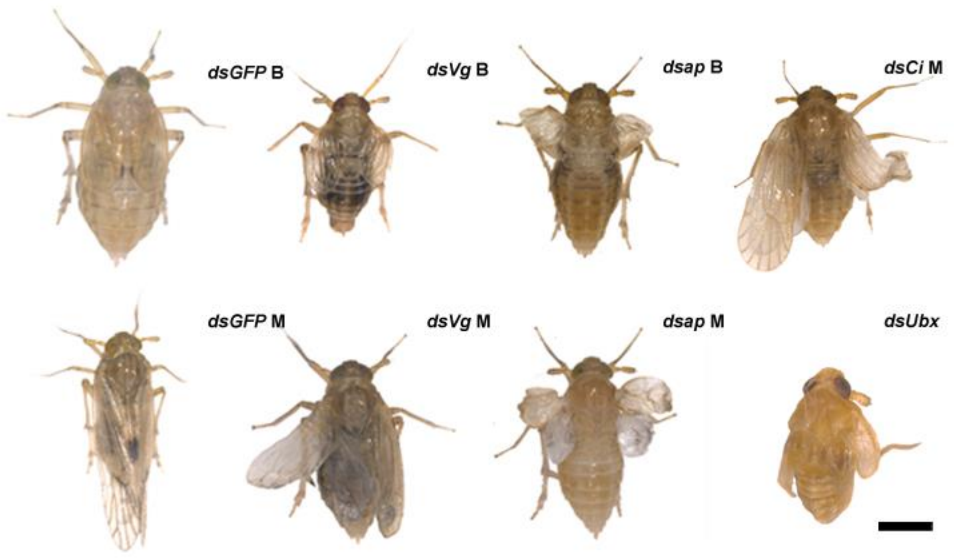

Figure S21. Genes involved in the wing developmental network in BPH

a. Differentially expressed wing development network genes revealed by qRT-PCR analysis of nymphs of various stages (first day of the 2nd, 3rd, 4th and 5th instars) feeding on yellow maturity rice compared to BPHs feeding on tillering rice. The red bars represent variations in relative gene expression level as compared to the lowest expressed gene. **b.** Phenotypes after RNAi treatments targeting *apterous* (*ap*), *vestigial* (*vg*), *cubitus interruptus* (*ci*) and *Ultrabithorax* (*Ubx*). B: brachypterous form; M: macropterous form. Scale bar = 1 mm.
